# LncRNA *LINC01503* promotes angiogenesis in colorectal cancer by regulating VEGFA expression *via* miR-342-3p and HSP60 binding

**DOI:** 10.7555/JBR.38.20240190

**Published:** 2024-10-22

**Authors:** Dandan Zheng, Xiya Zhang, Jia Xu, Shuwen Chen, Bin Wang, Xiaoqin Yuan

**Affiliations:** 1 Department of Anatomy, Histology and Embryology, Nanjing Medical University, Nanjing, Jiangsu 211166, China; 2 Jiangsu Key Laboratory of Oral Diseases, Nanjing Medical University, Nanjing, Jiangsu 210029, China; 3 Department of Clinical Medicine, First Clinical Medicine College, Nanjing Medical University, Nanjing, Jiangsu 210029, China; 4 Department of Gastroenterology, the Affiliated Changshu Hospital of Nantong University, Changshu No. 2 People's Hospital, Suzhou, Jiangsu 215500, China

**Keywords:** *LINC01503*, angiogenesis, VEGFA, colorectal cancer, HSP60, miR-342-3p

## Abstract

Colorectal cancer (CRC) ranks among the top five most common malignant tumors worldwide and has a high mortality rate. Angiogenesis plays an important role in CRC progression; however, anti-angiogenesis therapy still has many limitations. Long non-coding RNAs (lncRNAs) participate in tumor progression by regulating the expression of vascular endothelial growth factor in metastatic CRC. Thus, targeting specific lncRNAs may provide some new hope for anti-angiogenic strategies. Through analyzing data from both clinical samples and The Cancer Genome Atlas database, we found that the lncRNA *LINC01503* was specifically upregulated in CRC tissues and was associated with tumor progression and poor overall survival. We also demonstrated that *LINC01503* enhanced the capacity for tube formation and migration of vascular endothelial cells, thus promoting CRC tumorigenesis by upregulating vascular endothelial growth factor A (VEGFA) expression in CRC cells. Mechanistically, *LINC01503* promoted the expression of VEGFA by simultaneously regulating both mRNA and protein stability of VEGFA by binding to miR-342-3p and the chaperone HSP60, respectively. The upregulation of *LINC01503* in CRC cells was attributed to the CREB-binding protein CBP/p300-mediated H3K27 acetylation of the *LINC01503* promoter region. Taken together, our findings clarify the mechanism by which *LINC01503* may promote CRC angiogenesis, implying that *LINC01503* may serve as a potential prognostic biomarker and therapeutic target for CRC.

## Introduction

Colorectal cancer (CRC) is characterized by extremely high morbidity and mortality rates. According to Global Cancer Statistics 2022, CRC accounted for approximately 10.0% of all cancer cases and 9.4% of all cancer-related deaths worldwide^[1]^. The metastatic rate in early-stage CRC patients ranges from approximately 10% to 20%, but may reach up to approximately 50% for those with advanced stages^[2]^. The survival rates for localized versus distant CRC differ significantly. In the United States, patients with localized CRC have a five-year survival rate of approximately 91%, while those with distant CRC have a five-year survival rate of only approximately 15%^[3]^. Therefore, the pathogenesis and molecular mechanisms of CRC have become the focus of research to develop effective therapeutic agents with minimal side effects.

Neovascularization is an important channel for cancer cell metastasis, enabling cancer cells to reach distant metastatic sites *via* the blood vessels^[4]^. Angiogenesis is vital to sustaining tumor growth and facilitating metastasis, characterized by the proliferation and migration of vascular endothelial cells^[5]^. Thus, targeting the proliferation and migration of vascular endothelial cells is a potential strategy for CRC therapy^[6]^. Because vascular endothelial growth factor (VEGF) is a key mediator of vascular endothelial cell proliferation and migration^[7]^, anti-VEGF agents have been employed to treat metastatic CRC^[8]^. However, their use is constrained by a high risk of fatal bleeding complications, including severe side effects such as intestinal perforation and arterial embolism, limiting their widespread application in CRC treatment^[9–10]^. Therefore, identifying molecules that may be specifically targeted to inhibit tumor angiogenesis may offer significant clinical benefits to CRC patients.

Long non-coding RNAs (lncRNAs) are highly abundant RNA transcripts with limited or no protein-coding capacity^[11]^. They play crucial roles in immune responses, inflammation, and cancer progression through various pathways, such as serving as miRNA precursors and participating in DNA methylation, histone modification, and chromatin remodeling^[12–13]^. Moreover, highly expressed lncRNAs in tumors or blood may serve as diagnostic biomarkers in various cancers. Several lines of evidence have demonstrated that lncRNAs regulate angiogenesis, thereby influencing tumor initiation, development, and metastasis^[14–16]^. Specifically, lncRNAs such as *FLANC* and *PVT1* have been identified as key players in cancer progression by regulating VEGF expression in metastatic colorectal cancer^[17–18]^.

LncRNA *LINC01503* has been reported to be aberrantly expressed in tumors and to promote tumor progression^[19]^. However, whether *LINC01503* has an angiogenic effect remains unexplored. In the current study, we aimed to investigate the role and underlying mechanisms by which *LINC01503* may promote angiogenesis in CRC from human samples, as well as at both cellular and animal levels.

## Materials and methods

### Patients and clinical samples

In the current study, we collected 76 pairs of paraffin-embedded human CRC tissue samples and their corresponding adjacent normal mucosal samples at the First Affiliated Hospital of Nanjing Medical University. Patients were selected based on specific inclusion and exclusion criteria to ensure a well-defined and representative patient population. The inclusion criteria were as follows: (1) patients aged 45–80 years with histologically confirmed CRC; (2) no prior chemotherapy or radiotherapy before surgery; and (3) availability of sufficient tumor tissue samples for molecular analysis. The exclusion criteria were as follows: (1) patients with a history of other malignancies or significant comorbid conditions that might influence study outcomes; (2) patients who had undergone any surgical intervention or treatment that might alter normal tissue structure before sample collection. The clinicopathological features of CRC patients are summarized in ***Table 1***. These 76 pairs of CRC samples were immediately snap-frozen in liquid nitrogen after collection and stored at −80 ℃ until RNA extraction. This project was approved by the Institutional Review Board of Nanjing Medical University (Approval No. [2016] 640).

**Table 1:** The correlation between *LINC01503* expression levels and clinicopathological characteristics of colorectal cancer patients

Characteristics	Cases (*n*)	*LINC01503* expression		*P*-value
		Low (*n*)	High (*n*)	
Sex				0.245
Male	42	23	19	
Female	34	15	19	
Age (years)				0.080
≥60	39	16	23	
<60	37	22	15	
Tumor diameter (cm)				0.169
≥5	27	11	16	
<5	49	27	22	
Tumor location				0.500
Colon	41	21	20	
Rectum	35	17	18	
Clinical stage				0.040
Ⅰ/Ⅱ	38	21	17	
Ⅲ/Ⅳ	38	14	24	
T stage				0.031
Ⅰ	2	0	2	
Ⅱ	19	14	5	
Ⅲ	41	20	21	
Ⅳ	14	4	10	
N stage				0.323
Absent	37	20	17	
Present	39	18	21	
M stage				0.307
M0	72	37	35	
M1	4	1	3	
Vascular invasion				0.041
Absent	61	34	27	
Present	15	4	11	
Perineural invasion				0.500
Absent	67	34	33	
Present	9	4	5	
Analyzed with the Chi-square test. Abbreviations: T, tumor; N, node; M, metastasis.				

### Immunohistochemical staining and evaluation

Immunohistochemical staining was performed on paraffin-embedded tissue sections using anti-CD31 and vascular endothelial growth factor A (VEGFA) antibodies, as previously described^[20]^. Briefly, tissue specimens were paraffin-embedded and sliced into sections, which were then dewaxed with xylene and dehydrated with graded alcohol. The sections were heated in citrate buffer, followed by blocking with 3% H_2_O_2_ and sealing with goat serum. The sections were incubated with a primary antibody at 4 ℃ overnight, including anti-CD31 antibody (1∶200 dilution; Cat. #ab38624, Abcam, Cambridge, UK) and anti-VEGFA antibody (1∶200 dilution; Cat. #Ab171828, Abcam). Then, the sections were incubated with an HRP-labeled secondary antibody (Cat. #AFIHC0003, AiFang Biological, Hunan, China). The sections were treated with 3,3′-diaminobenzidine tetrahydrochloride reagent to develop color, and counterstained with hematoxylin. Each section was photographed under the microscope (Zeiss, Melville, NY, USA) at 200× magnification.

The VEGFA scores were analyzed using a modified H-score. Briefly, five fields at 400× magnification were randomly selected, and the staining intensity was rated as 0, 1, 2, or 3, corresponding to negative, low, moderate, and strong staining, respectively. The semi-quantitative assessment was performed by two independent investigators who were blinded to the clinical parameters of the patients examined. The H-score was calculated by the formula: [(% of weak staining) × 1] + [(% of moderate staining) × 2] + [(% of strong staining) × 3]^[21]^. The expression levels of VEGFA were then categorized as low or high based on the median H-score for each patient^[22]^.

After immunohistochemical staining with CD31 antibody, microvessels were quantified according to the method described by Weidner *et al*^[23]^. Single endothelial or clustered endothelial cells, with or without a lumen, were considered individual vessels. ImageJ software (National Institutes of Health, Bethesda, MD, USA) was used to quantify the microvessel density (MVD) based on the CD31 staining^[23]^.

### Cells and cell culture

The HCT116, RKO, Lovo, and Caco-2 CRC cell lines were obtained from the Shanghai Institute of Cell Biology of the Chinese Academy of Sciences. The CX-1 cell line was purchased from Fuheng Cell Center (Shanghai, China). The cell lines were authenticated by short tandem repeat profiling and confirmed to be free of *Mycoplasma* contamination. The cells were cultured in either RPMI 1640 or DMEM medium supplemented with 10% fetal bovine serum (FBS), 100 U/mL penicillin, and 0.1 mg/mL streptomycin (all reagents from Gibco, Grand Island, NY, USA), and incubated in a humidified incubator at 37 ℃ with 5% CO_2_.

Human umbilical vein endothelial cells (HUVECs) were isolated from the umbilical cords of healthy donors at Nanjing Maternal and Child Health Hospital (Approval No. PJ2021-039-001) by incubating the veins with 0.1% collagenase D (Roche, Mannheim, Germany) for 35 min. HUVECs were cultured in endothelial cell growth medium (Cat. #1001-b, ScienCell, Carlsbad, CA, USA), which was changed twice a week.

### Cell counting kit-8 (CCK8) assay

The CCK8 kit (Medchem Express, Monmouth Junction, NJ, USA) was used to evaluate cell proliferation. CRC cells were seeded in 96-well plates at a density of 1 × 10^3^ cells per well and then transfected with either small interfering RNA (siRNA) or plasmid. Absorbance at a wavelength of 450 nm was measured at different time points using a microplate reader (Bio-Tek, Winooski, VT, USA). Each experiment was performed in triplicate and repeated at least three times.

### Colony formation assay

Cells transfected with siRNA or plasmids were cultured in six-well plates at a density of 500 cells per well for 10 to 15 days, with the medium changed every three days. The colonies were stained using a 0.1% solution of crystal violet (Keygene, Nanjing, China), and then counted to determine the degree of colony formation. Each experiment was repeated at least three times.

### Collection of conditioned medium

CRC cells were transfected with either siRNA or plasmids, and the culture supernatant was removed 48 h later. The cells were then washed three times with phosphate-buffered saline (PBS), and cultured in a serum-free basal medium. After 48 h, the conditioned medium was collected, filtered through a 0.45-μm filter (Merck Millipore, Darmstadt, Germany), and stored at −80 ℃ for further use.

### Transwell assays

We performed transwell assays to evaluate cell migration and invasion using 24-well transwell chambers with an 8-μm pore size polycarbonate membrane (Corning, NY, USA). A total of 3 × 10^4^ CX-1 cells, 6 × 10^4^ RKO/HCT116 cells, and 1.6 × 10^4^ HUVECs in 200 μL of serum-free medium were seeded into the upper chamber, which was either coated with Matrigel (Corning) for invasion assays or left uncoated for migration assays. The lower chamber contained 600 μL of medium containing 10% FBS. For the assays involving HUVECs, the ratio of conditioned medium to basal medium was 2∶1. After an incubation period of 10 to 48 h, the non-migrating or non-invading cells on the upper surface were removed. Migrating and invading cells on the bottom were fixed with 95% ethanol, stained with 0.1% crystal violet in methanol/PBS, and imaged using a Zeiss microscope (Melville, NY, USA). In each group, five fields per filter were selected at random, and stained cells were counted to assess the degree of cell migration and invasion. Each experiment was repeated at least three times.

### HUVECs tube formation assay

Pre-cooled Matrix (Corning) was added to the wells of a 96-well plate and polymerized at 37 ℃ for 30 min. HUVECs (8 × 10^3^) were suspended in 100 μL medium (conditioned medium to basal medium ratio = 2∶1), and incubated at 37 ℃, 5% CO_2_ for 1 to 6 h. Quantitative analysis of the total length of the tubes in each well was performed using ImageJ software. Each experiment was repeated at least three times.

### Tumor formation and metastasis assays in nude model mice

Male BALB/c nude mice (4–5 weeks old) (GemPharmatech, Nanjing, China) were divided into two groups (*n* = 5 for each group). Mice were maintained under pathogen-free conditions with a 12-h light/12-h dark cycle. *LINC01503*-knockdown HCT116 cells or control cells (1 × 10^7^) were subcutaneously injected into the dorsal flank of mice. Tumor growth was evaluated every two days for four weeks. The tumor volume was measured using the following formula: volume = length × width^2^ × 0.5. After four weeks, all mice were euthanized, and the xenograft tumors were collected for further analysis.

For the *in vivo* lung metastasis assay, HCT116 cells with *LINC01503* stable knockdown and control cells (4 × 10^6^) were injected *via* the tail vein into each nude mouse (*n* = 5 per group). After eight weeks, the mice were euthanized, and their lungs were examined for metastases. The study protocols were approved by the Animal Care and Use Committee of Nanjing Medical University (Approval No. 1601080). All procedures strictly adhered to the Guide for the Care and Use of Laboratory Animals.

### Chick embryo chorioallantoic membrane (CAM) assay

Fertilized White Leghorn chicken eggs were incubated at 37 ℃ with constant humidity. On the 8th day of incubation, a small window was carefully created in the shell above the air sac, and then sealed with paraffin. CRC cells (1×10^7^ in 50 μL) were mixed with 50 μL medium containing 50% High Concentration Matrigel (BD Biosciences, San Jose, CA, USA). A 0.1 mL aliquot of this cell suspension was applied to the CAM of 8-day-old embryos. Four to five days after implantation, photographs of the Matrigel implants were taken, and blood vessel counts were conducted by two independent observers who were blinded to the treatment groups.

### siRNA, plasmid, and miRNA mimic/inhibitor transfection

Short interfering RNAs (siRNAs), plasmids, miRNA mimics, or miRNA inhibitors were transfected into cells using Lipofectamine 2000 transfection reagent (Invitrogen, Carlsbad, CA, USA), following the manufacturer's instructions. Briefly, 1 × 10^5^ cells were seeded in a 6-well plate and incubated for 24 h until reaching a confluence of approximately 40%, which was considered to be the optimal density for transfecting 4 μL of siRNA or miRNA mimics and inhibitors to a final concentration of 50 nmol/L for both siRNA or miRNA mimics and inhibitors. Meanwhile, seeding 3 × 10^5^ cells under the same conditions resulted in a confluence of about 80%, which was suitable for transfecting 4 μg of plasmids. Six hours after transfection, the medium was replaced with complete medium containing 10% FBS, and cells were cultured for an additional 24 to 48 h before further studies.

siRNAs targeting *LINC01503* (si-*LINC01503*-1, si-*LINC01503*-2, and si-*LINC01503*-3), *CBP* (si-*CBP*), and *P300* (si-*P300*), along with a negative control siRNA (si-NC), were purchased from RiboBio Co., Ltd. (Guangzhou, Guangdong, China). For overexpression experiments, the cDNAs of *LINC01503* and *HSP60* were cloned into the pcDNA3.1 expression vector to generate pcDNA-*LINC01503* and pcDNA-*HSP60* constructs, respectively, by GeneChem (Shanghai, China). Additionally, miRNA mimics and inhibitors specific to miR-342-3p were also obtained from RiboBio. The sequences for the siRNAs targeting *LINC01503*, *CBP*, and *P300* are shown in ***Supplementary Table 1*** (available online).

### Analysis of The Cancer Genome Atlas (TCGA)-CRC data

The transcriptome expression profiles of CRC tumor tissues and paired normal tissues, along with corresponding clinical data, were downloaded from the TCGA data portal (https://cancergenome.nih.gov). Specifically, we used data from the TCGA Colon Adenocarcinoma Collection (TCGA-COAD)^[24]^ to analyze differences in *LINC01503* expression between CRC and normal tissues. We downloaded TCGA CRC data in July 2020. Furthermore, to determine the correlation between *LINC01503* expression levels and the overall survival of CRC patients, Kaplan-Meier analysis was performed. Correlation analysis of *LINC01503* with VEGFA and HSP60 was also performed using GraphPad Prism 8.0 (GraphPad Software, San Diego, CA, USA).

### RNA extraction and real-time reverse transcription-PCR (qRT-PCR)

Total RNA (1 μg) was reversely transcribed into cDNA using the PrimeScript 1st Strand cDNA Synthesis Kit (Takara Bio, Shiga, Japan). For miRNA, the TaqMan MicroRNA Reverse Transcription Kit (Applied Biosystems, Foster City, CA, USA) and specific primers were used for reverse transcription. The expression levels of the respective RNAs were quantified using SYBR Green Master Mix (Takara Bio) on an ABI 7300 sequence detector (Applied Biosystems). Relative RNA expression levels were calculated using the ΔΔCt method, with *GAPDH* and *U6* serving as internal controls for mRNAs and miRNAs, respectively. The primers used for PCR are shown in ***Supplementary Table 2*** (available online). Each experiment was repeated at least three times.

### Enzyme-linked immunosorbent assay (ELISA)

Cells were transfected according to experimental grouping for 48 h and then cultured in serum-free basal medium for an additional 24 h. The supernatant was collected and filtered through a 0.45-μm filter. VEGFA was detected using a Human VEGFA ELISA kit (Cat. #EHC108.96, NeoBioscience, Shenzhen, China) following the manufacturer's protocol. First, the supernatant was added for incubation and washing, followed by the addition of a biotinylated antibody for incubation and washing. Next, the enzyme conjugate was added for incubation and washing. Finally, the chromogenic substrate was added for incubation, and the reaction was terminated with a stop solution. Absorbance was measured at 450 nm using a microplate reader (Bio-Tek).

### Generation of stable *LINC01503* knockdown cells

Lentiviruses carrying sh-*LINC01503* or scrambled shRNA were constructed by Genechem (Shanghai, China). HCT116 cells were infected with these lentiviruses at a multiplicity of infection of 20. After infection, stable transductants were selected using 4 μg/mL puromycin (Sigma, St. Louis, MO, USA). The sequence for the shRNA targeting *LINC01503* was 5′-CCACCTTTCTGGTAATGCA-3′.

### Western blotting (WB) analysis

Lysates from the cells or tumor tissues were separated by SDS-PAGE and then transferred onto polyvinylidene fluoride membranes. The membranes were subsequently incubated with primary antibodies, followed by incubation with appropriate secondary antibodies. Protein detection was carried out using a chemiluminescent method. Antibodies against the following antigens were used in the current study: VEGFA (1∶1000 dilution; Cat. #Ab71828, Abcam, Cambridge, UK), HSP60 (1∶1000 dilution; Cat. #SC-271215, Santa Cruz, CA, USA), AKT (1∶1000 dilution; Cat. #4691, Cell Signaling Technology, Danvers, MA, USA), p-AKT (Thr308) (1∶1000 dilution; Cat. #13038, Cell Signaling Technology), PCNA (1∶1000 dilution; Cat. #SC-56, Santa Cruz), Histone H3 (1∶1000 dilution; Cat. #D1H2, Cell Signaling Technology), histone H3 lysine 27 acetylation (1∶1000 dilution; Cat. #Ab4729, Abcam), CBP (1∶1000 dilution; Cat. #Ab253202, Abcam), p300 (1∶1000 dilution; Cat. #Ab275378, Abcam), and GAPDH (1∶2000 dilution; Cat. #SC-47724, Santa Cruz). Each experiment was repeated at least three times.

### Dual-luciferase reporter assay

Sequences of *LINC01503* with predicted miR-342-3p binding sites, along with their respective mutated sequences, were amplified by PCR and cloned into the pmirGLO vector (Genechem) to create LINC-WT (5′-GTGCAGGGATTACAGGTGTGAGC-3′) and LINC-MUT (5′-CCAGAGACCCTGAGCCCAGAGAA-3′) plasmids, respectively. The same procedure was used to construct the *VEGFA* plasmids. These plasmids were co-transfected into CRC cells along with either a miR-342-3p mimic or a miR-342-3p mimic negative control (mimic-NC). Dual-luciferase assays (Promega, Madison, WI, USA) were then performed following the manufacturer's protocol. Each experiment was repeated at least three times.

### Immunoprecipitation (IP)

RKO and CX-1 cells were lysed with IP lysis buffer (Beyotime Biotech, Shanghai, China). We incubated 20 μL Protein A/G beads (Sigma-Aldrich) with anti-HSP60 (2 μg) or control IgG (2 μg) overnight at 4 ℃. The cell lysate (total 1 mg) was added to the antibody-bead complex and rotated at 4 ℃ overnight. After washing, 1× SDS sample buffer (30 μL) was added to the antibody-bead complex and heated at 95 ℃ for 10 min to elute the target protein. Elution products were analyzed by WB with anti-HSP60 (Santa Cruz) and anti-VEGFA (Abcam). HCT116 cells were transfected with pcDNA-HSP60-Flag (GeneChem), and pcDNA3.1 was used as a negative control. After 48 h of transfection, the cells were lysed with IP lysis buffer (Beyotime Biotech). Then, cell lysates (total 1 mg protein) were incubated with 20 μL anti-Flag magnetic beads (Cat. M8823, Sigma-Aldrich) overnight at 4 ℃. After incubation, the beads were washed with lysis buffer. The precipitated proteins were eluted with 1× SDS sample buffer and analyzed by WB with anti-Flag (Sigma-Aldrich), anti-HSP60 (Santa Cruz), and anti-VEGFA (Abcam).

### RNA immunoprecipitation (RIP)

The RIP experiment was performed using the EZ-Magna RIP Kit (Millipore, MA, USA) according to the manufacturer's instructions. In brief, HCT116 cells were transfected with either a miR-342-3p mimic or a miR-342-3p mimic-NC for 24 h, and 1 × 10^7^ cells were pelleted and resuspended in RIP lysis buffer. The lysates were incubated with magnetic beads conjugated to IgG (5 μg; Millipore) or the AGO2 antibody (5 μg; Cat. #ab186733, Abcam) and rotated at 4 ℃ overnight. Similarly, 1 × 10^7^ untreated HCT116 and CX-1 cells were pelleted and resuspended in RIP lysis buffer. The lysates were incubated with IgG (5 μg; Millipore) and the anti-HSP60 (5 μg; Cat. #ab19082, Abcam) antibody-coated magnetic beads and rotated at 4 ℃ overnight. The immunoprecipitated proteins were digested with proteinase K. The purified RNA was then subjected to qRT-PCR to examine the levels of *LINC01503* or *VEGFA*. Each experiment was repeated at least three times.

### Chromatin immunoprecipitation (ChIP)

The ChIP assay was performed using a ChIP kit (Abcam) according to the manufacturer's instructions. Briefly, 3 × 10^6^ cells were fixed with formaldehyde, lysed, and sonicated to shear the DNA into fragments ranging from 200 to 1000 bp. The DNA fragments were immunoprecipitated using 5 μg of an anti-H3K27ac antibody (Abcam) or a negative-control IgG (Millipore). The precipitated DNA was purified and then analyzed by qPCR. The primers used in the ChIP assay are shown in ***Supplementary Table 3*** (available online).

### Chromatin isolation by RNA purification (ChIRP) assay and mass spectrometry

The ChIRP assay was performed according to a previously described protocol^[25]^. Briefly, HCT116 cells were cross-linked with 1% formaldehyde for 10 min, equilibrated with glycine buffer for 5 min, washed thrice with cold PBS, and then scraped using 1 mL of lysis buffer. Cell lysates were sonicated to generate 200 to 1000 bp RNA fragments and subsequently centrifuged at 10000 *g* for 10 min. Next, 50 μL of the supernatant was transferred as the input. The remaining supernatant lysate was incubated with 3 μL of probes (100 μmol/L) at 37 ℃ with shaking. Samples were thoroughly mixed with 100 μL of prepared beads and incubated with rotation. The bead-sample mixture was washed twice with wash buffer and then resuspended in 1 mL of wash buffer. Next, 100 μL of the suspension was set aside for RNA isolation using TRIzol (Invitrogen). The remaining 900 μL of the suspension was centrifuged at 12 000 *g* for 10 min, and the pellet was resuspended in 50 μL of protein buffer and boiled for mass spectrometry analysis (performed by Shanghai Applied Protein Tech, Shanghai, China). The probes and primers used are shown in ***Supplementary Table 4*** (available online).

### Statistical analysis

Each experiment was repeated at least three times, and the means were used for analysis. Data were presented as the mean ± standard deviation. Statistical analysis was performed using SPSS 20.0 (IBM Corp., Armonk, USA). To compare the statistical differences between two groups, a Student's *t*-test was used. To compare the statistical differences among multiple groups, one-way ANOVA, followed by Dunnett's tests for multiple comparisons, was employed. The correlation between *LINC01503* levels and clinicopathological characteristics in CRC patients was analyzed using either the Chi-square test or Fisher's exact test. Cox proportional hazards regression models were used to calculate hazard ratios with 95% confidence intervals for univariable and multivariate analyses. Survival curves were plotted using the Kaplan-Meier method and compared using a log-rank test. A *P* < 0.05 was considered statistically significant.

## Results

### *LINC01503* expression was highly expressed and associated with an advanced stage, vascular invasion, and poor survival of CRC patients

To identify lncRNAs potentially involved in CRC progression, we collected primary tumors and corresponding adjacent normal mucosal tissues from three patients with stage Ⅰ/Ⅱ and three patients with stage Ⅲ/Ⅳ CRC, and performed an lncRNA microarray analysis^[25]^. The results revealed that *LINC01503* was not only expressed at a higher level in tumors than in normal tissues, but was also significantly increased in stage Ⅲ/Ⅳ CRC cases compared with stage Ⅰ/Ⅱ CRC cases (***Supplementary Fig. 1A***, available online). This finding indicates that *LINC01503* may be involved in the progression of CRC.

**Figure 1:**
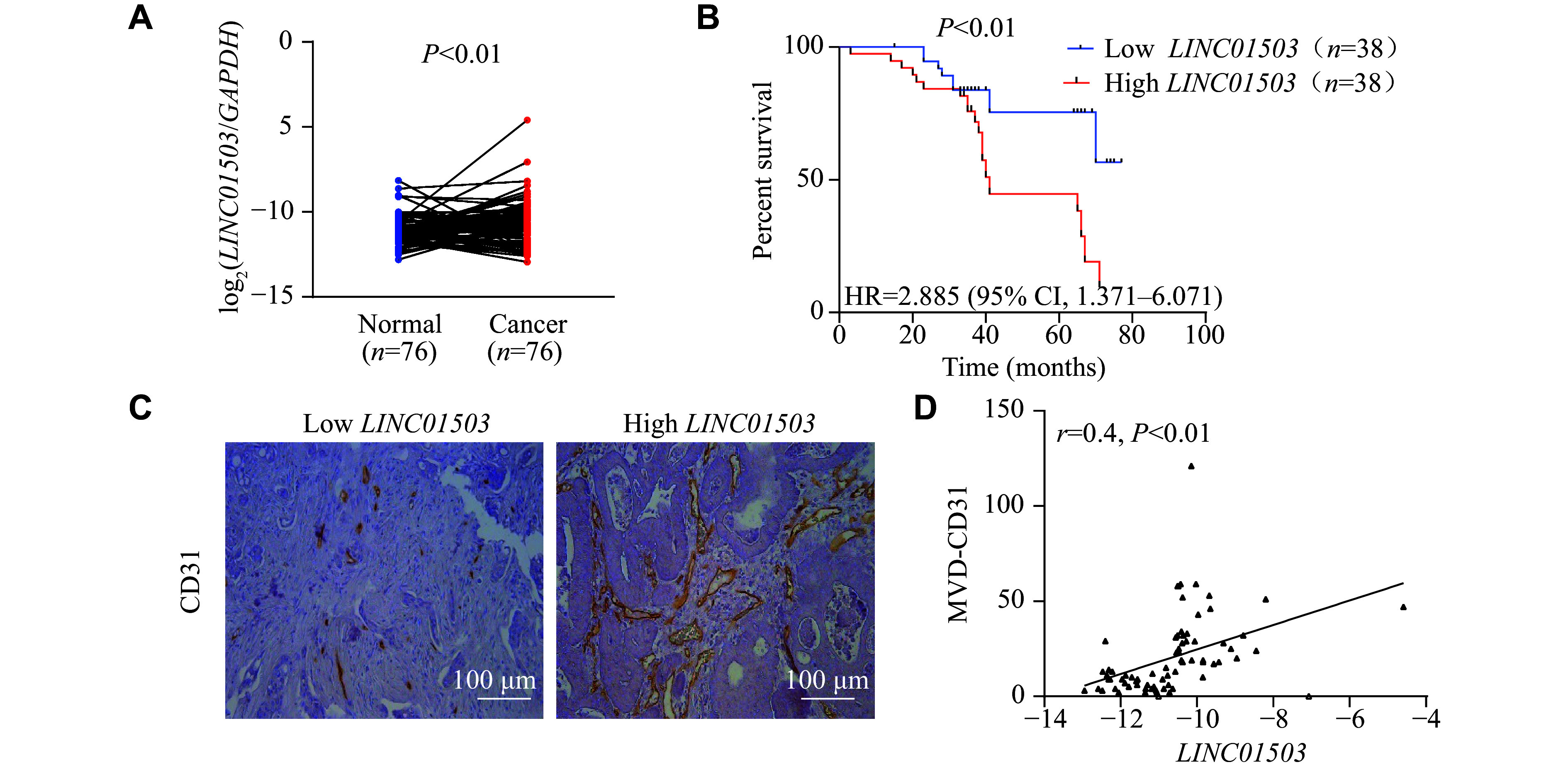
*LINC01503* was specifically overexpressed in colorectal cancer (CRC) tissues and was associated with poor prognosis. A: The expression levels of *LINC01503* in 76 pairs of matched clinical CRC samples were evaluated by qRT-PCR. *GAPDH* was used as an internal reference gene. *P* < 0.01 by paired Student's *t*-test. B: Kaplan-Meier survival curve of overall survival in CRC patients with low or high *LINC01503* expression levels. *P* < 0.01 by log-rank test. C: Representative images of CD31 immunohistochemistry in clinical CRC samples with low or high *LINC01503* expression levels. The expression of CD31 protein was evaluated in terms of MVD in the tumor tissues of the two groups (*n* = 38 for each group). D: The correlation between the expression levels of *LINC01503* and CD31^+^ MVD was analyzed. Abbreviations: qRT-PCR, real-time reverse transcription-PCR; MVD, microvessel density; HR, hazard ratio; CI, confidence interval.

To verify the results of the microarray analysis, we measured the expression levels of *LINC01503* in 76 pairs of matched CRC tumor tissues and normal colorectal tissues by qRT-PCR analysis. The results demonstrated that the expression levels of *LINC01503* were significantly increased in CRC tissues compared with normal colorectal tissues (*P* < 0.01; ***Fig. 1A***). We divided 76 CRC patients into LINC01503 high and low expression groups based on the median *LINC01503* expression. Further Kaplan-Meier survival analysis showed that CRC patients with high *LINC01503* expression levels had a shorter overall survival than those with low *LINC01503* expression levels (***Fig. 1B***). These results were consistent with the analysis outcomes from The Cancer Genome Atlas (TCGA) database (***Supplementary Fig. 1B***–***1D***, available online).

To investigate the clinicopathological significance of *LINC01503* in CRC, we analyzed the correlation between the expression levels of *LINC01503 *and the clinicopathologic characteristics of our CRC patient cohort. The results presented in ***Table 1*** revealed that high expression levels of *LINC01503 *were significantly correlated with clinical stage (*P* = 0.04), T stage (*P* = 0.031), and vascular invasion (*P* = 0.041). We further analyzed the correlation between *LINC01503* expression levels and angiogenesis in CRC tissues, and found that high expression levels of *LINC01503 *were significantly correlated with high MVDs (***Fig. 1C*** and ***1D***). Consistent with this finding, a positive correlation was found between the expression levels of *LINC01503* and *CD31* based on the TCGA data (***Supplementary Fig. 1E***, available online). Collectively, these results indicate that high expression of *LINC01503* in CRC tissues may promote disease progression and is associated with tumor angiogenesis.

### *LINC01503* promoted tumorigenesis in CRC

**Figure 2:**
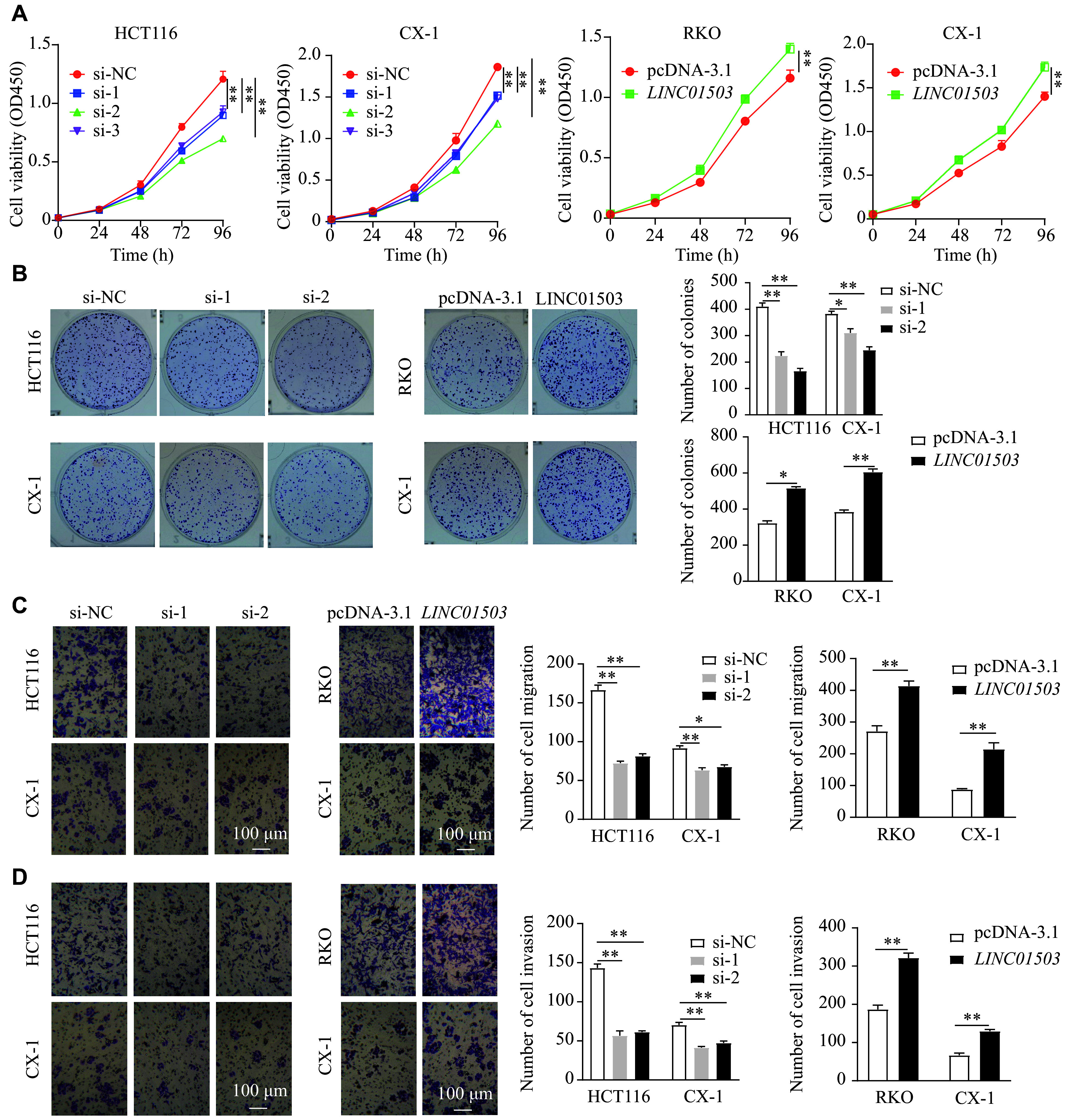
*LINC01503* promoted cell proliferation and invasion *in vitro*. HCT116 and CX-1 cells were transfected with *LINC01503* siRNAs (si-1, si-2, and si-3) or negative control siRNA (si-NC). RKO and CX-1 cells were transfected with *LINC01503* overexpressing plasmid or vector plasmid (pcDNA-3.1). Cell function experiments were performed 48 h after siRNA transfection and 24 h after plasmid transfection, respectively. A: Cell viability was assessed using the CCK-8 assay and detected at an optical density of 450 nm (OD450). B: Colony formation was assessed. C and D: Transwell assays were performed to detect cell migration (C) and invasion (D) of the transfected cells. Data are presented as mean ± standard deviation (*n* = 3). Two-way ANOVA followed by Dunnett's test (A) and two-tailed Student's *t*-test (B–D) were used to determine the significance of the difference between the groups. Abbreviations: CRC, colorectal cancer; siRNA, short interfering RNA; CCK-8, cell counting kit-8.

To investigate the biological function of *LINC01503* in CRC, we transfected CRC cell lines HCT116 and CX-1 with siRNAs targeting *LINC01503*, and cell lines RKO and CX-1 with pcDNA-*LINC01503* plasmids. The siRNA transfection efficiently knocked down the expression of *LINC01503*, while the pcDNA-*LINC01503* transfection led to an increase in *LINC01503* levels (***Supplementary Fig. 2A***, available online). The CCK-8 and colony formation assays demonstrated that, compared with the controls, silencing *LINC01503* significantly reduced the proliferative capacity of CRC cells, while overexpressing *LINC01503* enhanced the viability of CRC cells (***Fig. 2A*** and ***2B***). Additionally, the transwell assays revealed that, compared with controls, *LINC01503* knockdown significantly impaired the migration and invasion abilities of CRC cells, whereas *LINC01503* overexpression promoted these processes (***Fig. 2C*** and ***2D***).

**Figure 3:**
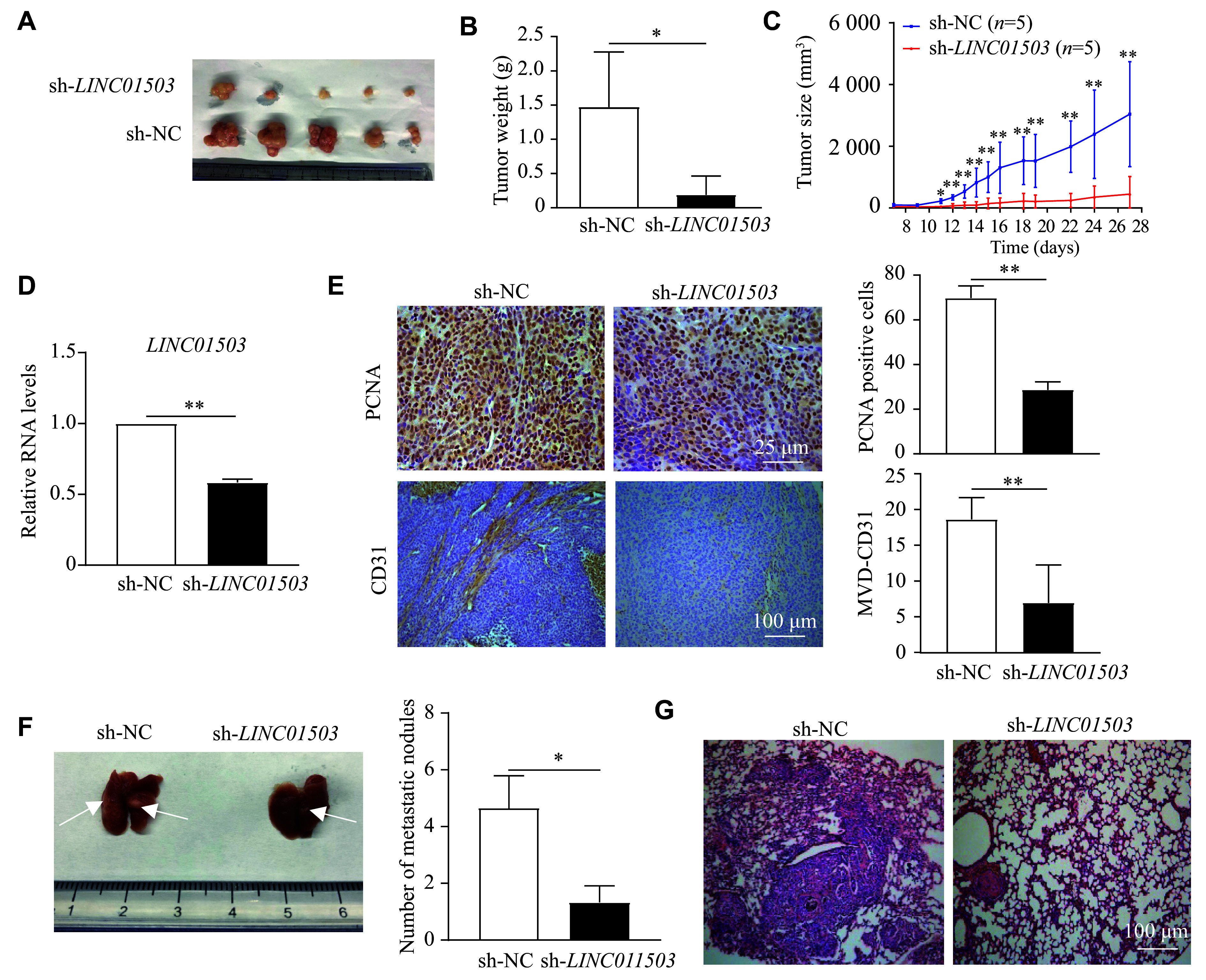
*LINC01503* promoted cell proliferation and invasion *in vivo*. A: Photographs of xenograft tumors from groups of BALB/c-nude mice at 28 days after injection with stable *LINC01503*-knockdown (sh-*LINC01503*) or control (sh-NC) HCT116 cells (*n* = 5). B: The subcutaneous tumor weights of nude mice were compared between the two groups (*n* = 5). C: The tumor growth curve of nude mice was plotted based on tumor volume in the two groups (*n* = 5). D: The expression levels of *LINC01503* in xenograft tumor tissues were evaluated by qRT-PCR (*n* = 5). E: Representative images of PCNA and CD31 immunohistochemistry in xenograft tumor tissues. PCNA and CD31 protein expression were assessed by immunohistochemistry assay (*n* = 5). F: Representative images of tumor metastasis in lung tissues (left) and the corresponding quantification of the number of metastatic foci in lung tissues (right) after tail vein injection with stable *LINC01503*-knockdown (sh-*LINC01503*) or control (sh-NC) HCT116 cells. The white arrows indicate the metastases (*n* = 5). G: Hematoxylin and eosin staining of lung tissues. Data are presented as mean ± standard deviation. ^*^P < 0.05 and ^**^P < 0.01 by unpaired Student's *t*-test. Abbreviations: CRC, colorectal cancer; qRT-PCR, real-time reverse transcription-PCR.

To determine the effect of *LINC01503* on tumorigenesis *in vivo*, we constructed the stable *LINC01503*-knockdown HCT116 cells (sh-*LINC01503*) and control HCT116 cells (sh-NC). In the xenograft experiments, nude mice inoculated with sh-*LINC01503* cells exhibited significantly lower tumor weights and volumes than the control mice (***Fig. 3A***–***3D***). Notably, silencing *LINC01503* significantly reduced the expression levels of *PCNA* and CD31^+^ MVD in tumor tissues (***Fig. 3E***). We next examined the effects of *LINC01503* on CRC metastasis by injecting cancer cells into mice *via* the tail veins. The results showed that silencing *LINC01503* significantly suppressed the formation of pulmonary metastatic nodules in the mice (***Fig. 3F*** and ***3G***). Collectively, these results indicate that *LINC01503* may promote CRC tumorigenesis.

### *LINC01503* promoted angiogenesis by increasing VEGFA expression

**Figure 4:**
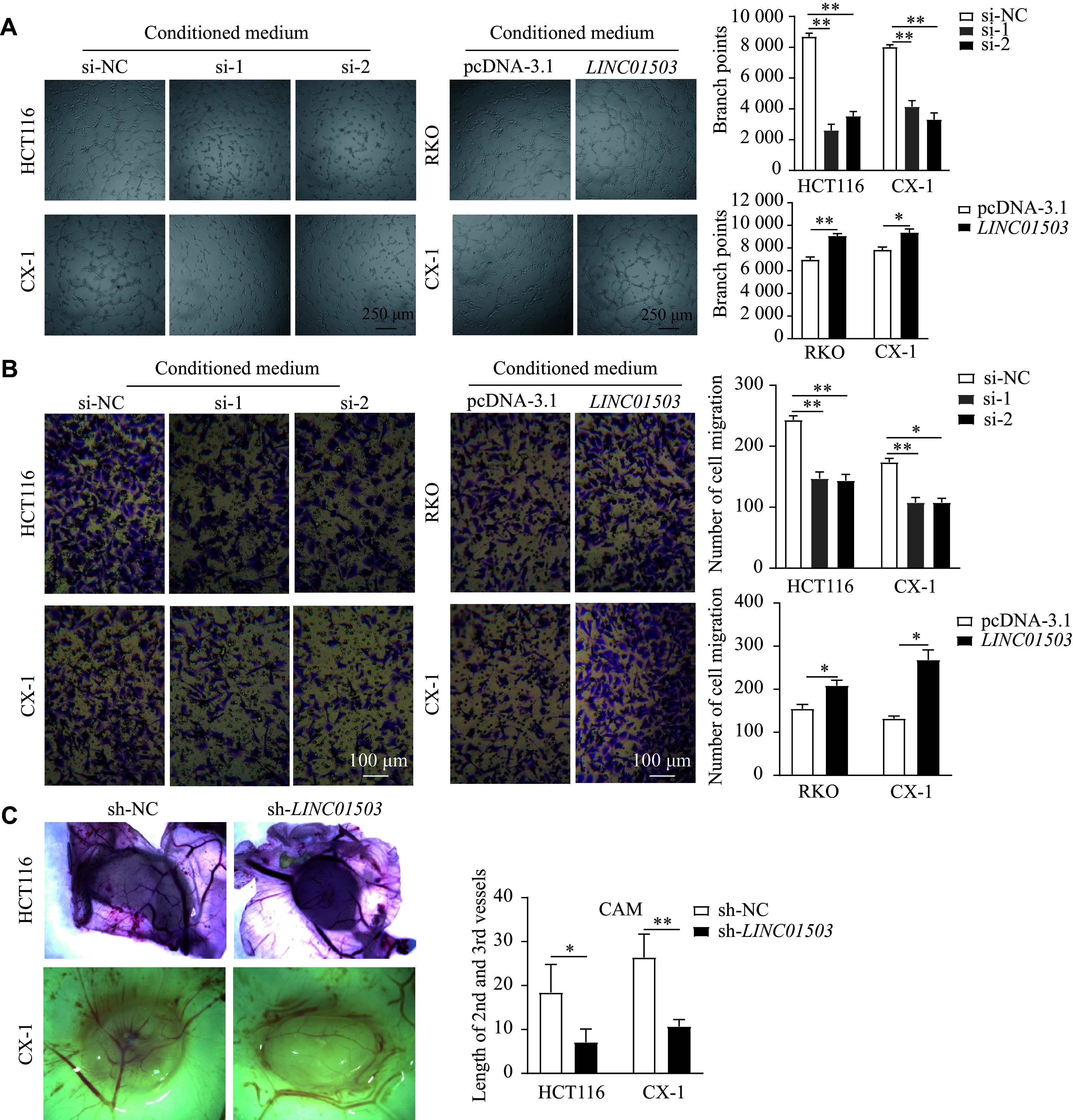
*LINC01503* promoted the proliferation and migration of vascular endothelial cells. After *LINC01503* knockdown or overexpression for 48 h in HCT116, CX-1, and RKO cells, the medium was replaced with a serum-free medium. Conditioned media were collected after an additional incubation of 24 h. A and B: The effects of conditioned medium from CRC cells with different *LINC01503* expression levels on the formation of branch points (A) and the migration ability (B) of HUVECs were evaluated by tube formation and transwell assay, respectively. C: The effects of *LINC01503* on the lengths of secondary and tertiary vessels were analyzed by the chick embryo chorioallantoic membrane assay. Data are presented as mean ± standard deviation (*n* = 3). Statistical significance was determined by one-way ANOVA, followed by Dunnett's test for multiple comparisons and unpaired Student's *t*-test. ^*^P < 0.05 and ^**^P < 0.01. Abbreviations: CRC, colorectal cancer; HUVECs, human umbilical vein endothelial cells.

To investigate the function of *LINC01503* in angiogenesis, we performed tube formation and transwell assays using HUVECs. The results showed that conditioned medium from *LINC01503*-knockdown CRC cells significantly reduced the branch points formed by HUVECs and impaired their migration ability, compared with that from the control cells. In contrast, the conditioned medium from *LINC01503*-overexpressing CRC cells significantly increased the branch points formed by HUVECs and enhanced their migration ability, compared with that from the control cells (***Fig. 4A*** and ***4B***). Subsequently, the pro-angiogenic effect of *LINC01503* was verified by the chick embryo CAM assay. The results demonstrated that silencing *LINC01503* led to a reduction in the length of the second and third vessels of CRC cells cultured on the CAM (***Fig. 4C***). These results indicate that *LINC01503* may have a pro-angiogenic function.

**Figure 5:**
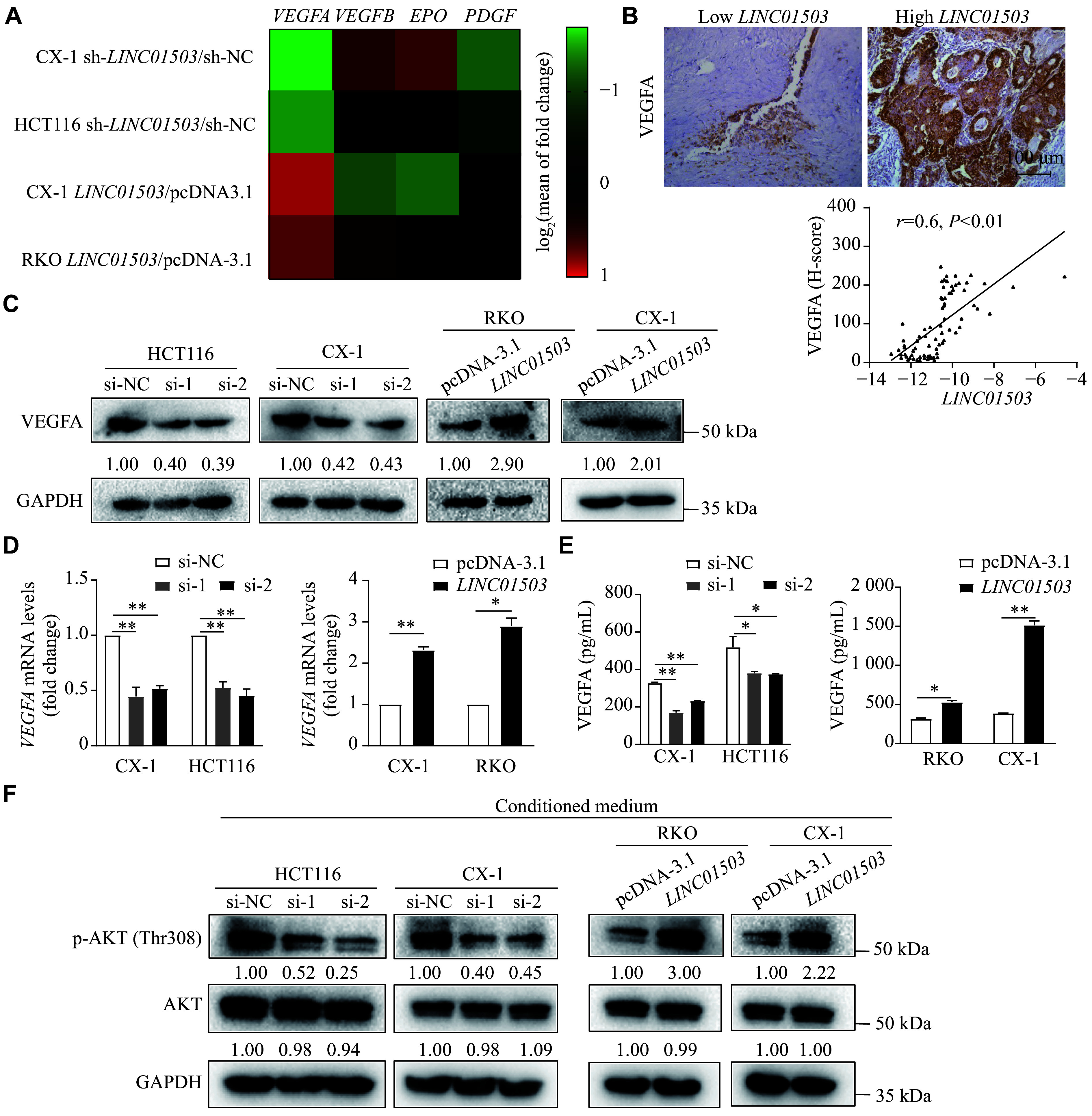
*LINC01503* induced vascular endothelial growth factor A (VEGFA) expression and secretion. A: HCT116 and CX-1 cells were infected with *LINC01503* knockdown lentiviruses, while RKO and CX-1 cells were transfected with *LINC01503* overexpression plasmids. qRT-PCR analysis was conducted to assess the expression of multiple angiogenesis-associated genes, with pseudo-color scale values log_2_ transformed. Transcript levels were normalized to *ACTB*. B: Representative images of VEGFA immunohistochemistry in clinical colorectal cancer samples with high or low *LINC01503* expression are shown. The correlation between *LINC01503* and VEGFA expression levels was analyzed. C: Western blotting (WB) analysis demonstrated the effect of *LINC01503* on VEGFA protein levels. D: qRT-PCR analysis assessed the effect of *LINC01503* expression on *VEGFA* mRNA levels. E: The effect of *LINC01503* on VEGFA secretion from CRC cells was analyzed by enzyme-linked immunosorbent assay. F: Following 48 h of *LINC01503* knockdown or overexpression in HCT116, CX-1, and RKO cells, the medium was replaced with a serum-free medium. Conditioned media were collected after an additional 24 h. The effect of supernatant from CRC cells with high or low *LINC01503* expression on the Akt signaling pathway in HUVECs was evaluated by WB. Data are presented as mean ± standard deviation (*n* = 3). Statistical significance was determined by one-way ANOVA, followed by Dunnett's test for multiple comparisons and unpaired Student's *t*-test. ^*^P < 0.05 and ^**^P < 0.01. Abbreviations: HUVECs, human umbilical vein endothelial cells; qRT-PCR, real-time reverse transcription-PCR; VEGFA, vascular endothelial growth factor A.

qRT-PCR analysis showed that, among the cell proliferation and migration-related genes expressed in vascular endothelial cells, the expression level of *VEGFA* was the most significantly correlated with the variations in *LINC01503* levels (***Fig. 5A***). We also found that the expression levels of *LINC01503* were significantly correlated with the protein levels of VEGFA in CRC tissues (***Fig. 5B***). This finding was consistent with the results of the analysis of TCGA data, which showed that the expression levels of *LINC01503* and *VEGFA* were significantly correlated (***Supplementary Fig. 2B***, available online). The WB and qRT-PCR results demonstrated that the overexpression of *LINC01503* upregulated both protein and mRNA expression levels of VEGFA, whereas knockdown of *LINC01503* exhibited the opposite effect (***Fig. 5C*** and ***5D***). The ELISA results also showed that the protein levels of VEGFA in the supernatant of CRC cells were significantly affected by changes in *LINC01503* expression (***Fig. 5E***), displaying a consistent trend with that observed in CRC cells.

The VEGFA-AKT pathway is well known to promote endothelial cell proliferation and new blood vessel formation. Therefore, we stimulated HUVECs with conditioned medium from CRC cells with low or high *LINC01503* expression levels. The results demonstrated that the conditioned medium from CRC cells with high *LINC01503* expression significantly activated the AKT signaling pathway in HUVECs, whereas the conditioned medium from CRC cells with low *LINC01503* expression inhibited the AKT signaling pathway in these cells (***Fig. 5F***).

Taken together, these findings indicate that *LINC01503* may increase the capacity for tube formation and migration of vascular endothelial cells by promoting the expression and secretion of VEGFA.

### *LINC01503* promoted VEGFA expression by binding to miR-342-3p

**Figure 6:**
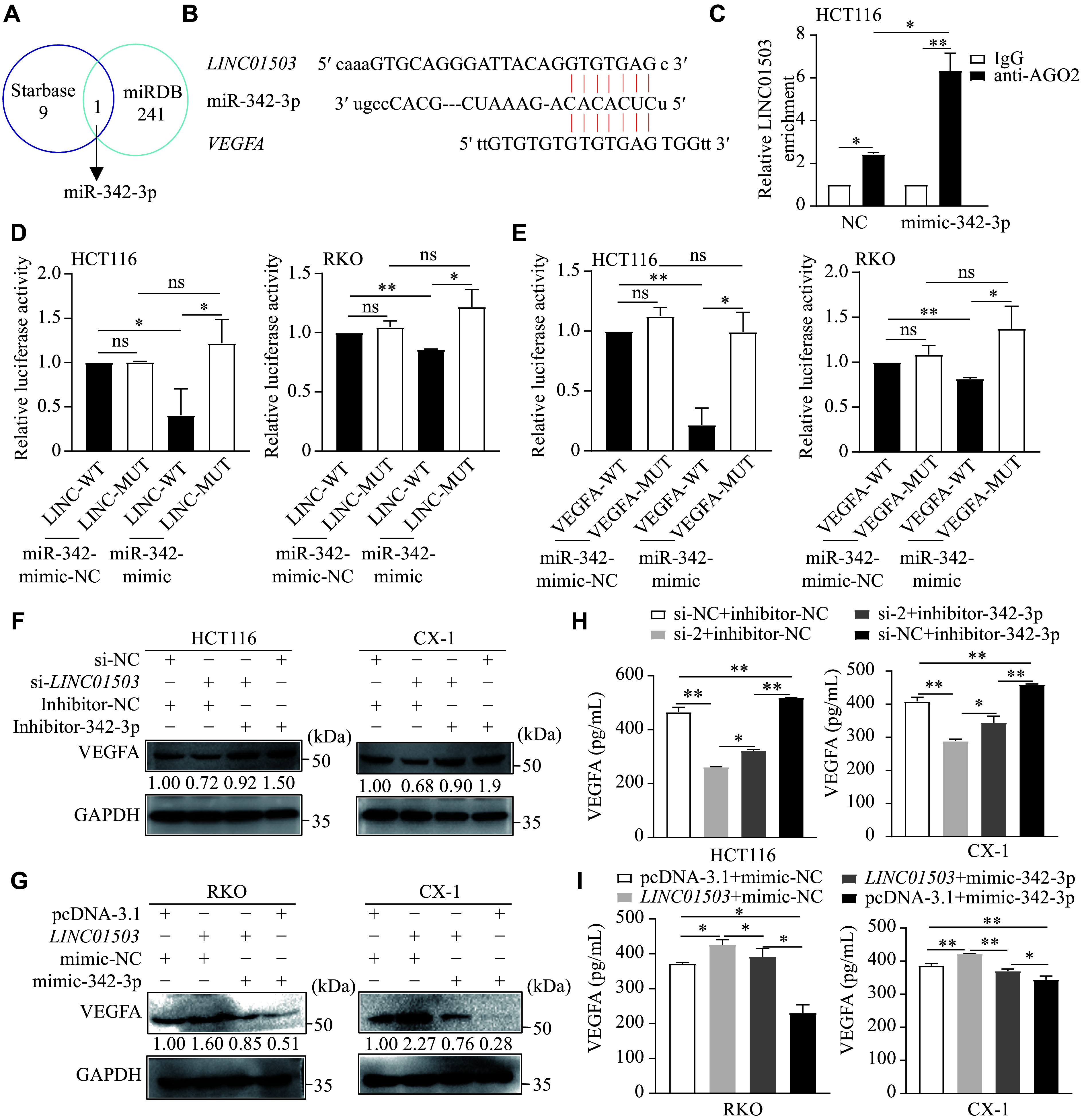
*LINC01503* promoted VEGFA expression by binding to miR-342-3p. A: Predicted miRNAs that bind to *LINC01503* were identified using the miRDB and Starbase databases. B: Sequences of miR-342-3p predicted to target both *LINC01503* and VEGFA are shown. C: RNA immunoprecipitation (RIP) was performed in HCT116 cells transfected with the miR-342-3p mimic or mimic-NC, using anti-IgG or anti-Ago2 antibodies; and the levels of *LINC01503* were quantified by qRT-PCR. D: HCT116 and RKO cells were co-transfected with the miR-342-3p mimic or mimic-NC and a reporter plasmid containing either the wild-type (LINC-WT) or mutant *LINC01503* binding sequence. Luciferase reporter assays measured the activity. E: HCT116 and RKO cells were similarly co-transfected with the miR-342-3p mimic or mimic-NC and a luciferase reporter plasmid containing either the wild-type (VEGFA-WT) or mutant VEGFA 3′ UTR (VEGFA-MUT). Relative luciferase activity assays assessed the activity. F and G: Western blotting was performed to measure VEGFA levels after co-transfection of the miR-342-3p inhibitor with si-*LINC01503* in HCT116 and CX-1 cells (F), or the miR-342-3p mimic with the *LINC01503* overexpression plasmid in RKO and CX-1 cells (G). H and I: ELISA was used to quantify secreted VEGFA levels following co-transfection of the miR-342-3p inhibitor with si-*LINC01503* in HCT116 and CX-1 cells (H), or the miR-342-3p mimic with the *LINC01503* overexpression plasmid in RKO and CX-1 cells (I). Data are presented as mean ± standard deviation (*n* = 3). ^*^P < 0.05 and ^**^P < 0.01 by one-way analyses of variance, followed by Dunnett's tests for multiple comparisons and unpaired Student's *t*-test. Abbreviations: CRC, colorectal cancer; qRT-PCR, real-time reverse transcription-PCR; VEGFA, vascular endothelial growth factor A.

We next investigated how *LINC01503* increased VEGFA expression. LncRNAs may promote the expression of related mRNAs by binding to miRNAs. We therefore used bioinformatics to predict miRNAs that bind to *LINC01503* using miRDB (http://mirdb.org/) and Starbase (https://starbase.sysu.edu.cn/), and selected miRNAs with binding scores ≥ 60. We found that miR-342-3p was the only miRNA that overlapped between the two databases (***Fig. 6A***). Analysis of the miRanda algorithms revealed that both *LINC01503* and *VEGFA* contained miR-342-3p-binding sequences (***Fig. 6B***). We then speculated that *LINC01503* might promote VEGFA expression by binding to miR-342-3p. The following RIP assays demonstrated a direct interaction between miR-342-3p and the *LINC01503* transcript (***Fig. 6C***). Furthermore, a luciferase reporter assay showed that upregulating miR-342-3p inhibited the luciferase reporter activity of *LINC01503*, but not that of mutant-*LINC01503* (***Fig. 6D***). Similar results were observed for *VEGFA* (***Fig. 6E***). These results suggested that *LINC01503* bound to miR-342-3p, and that miR-342-3p bound to *VEGFA* mRNA at the same site. Significantly, the miR-342-3p mimic abolished the tube formation and migration abilities of HUVECs induced by *LINC01503* overexpression in CRC cells. Conversely, a miR-342-3p inhibitor reversed the inhibitory effects on the tube formation and migration capacities of HUVECs induced by *LINC01503* knockdown in CRC cells (***Supplementary Fig. 3A–3D***, available online). Moreover, the miR-342-3p mimic abrogated the increased expression and secretion of VEGFA induced by *LINC01503* overexpression in CRC cells, while the miR-342-3p inhibitor reversed the decreased VEGFA expression and secretion induced by *LINC01503* knockdown in CRC cells (***Fig. 6F***–***6I***).

These results indicate that *LINC01503* promotes VEGFA expression and secretion by binding to miR-342-3p, thereby increasing the tube formation and migration ability of HUVECs.

### *LINC01503* enhanced the protein stability of VEGFA by binding to HSP60

**Figure 7:**
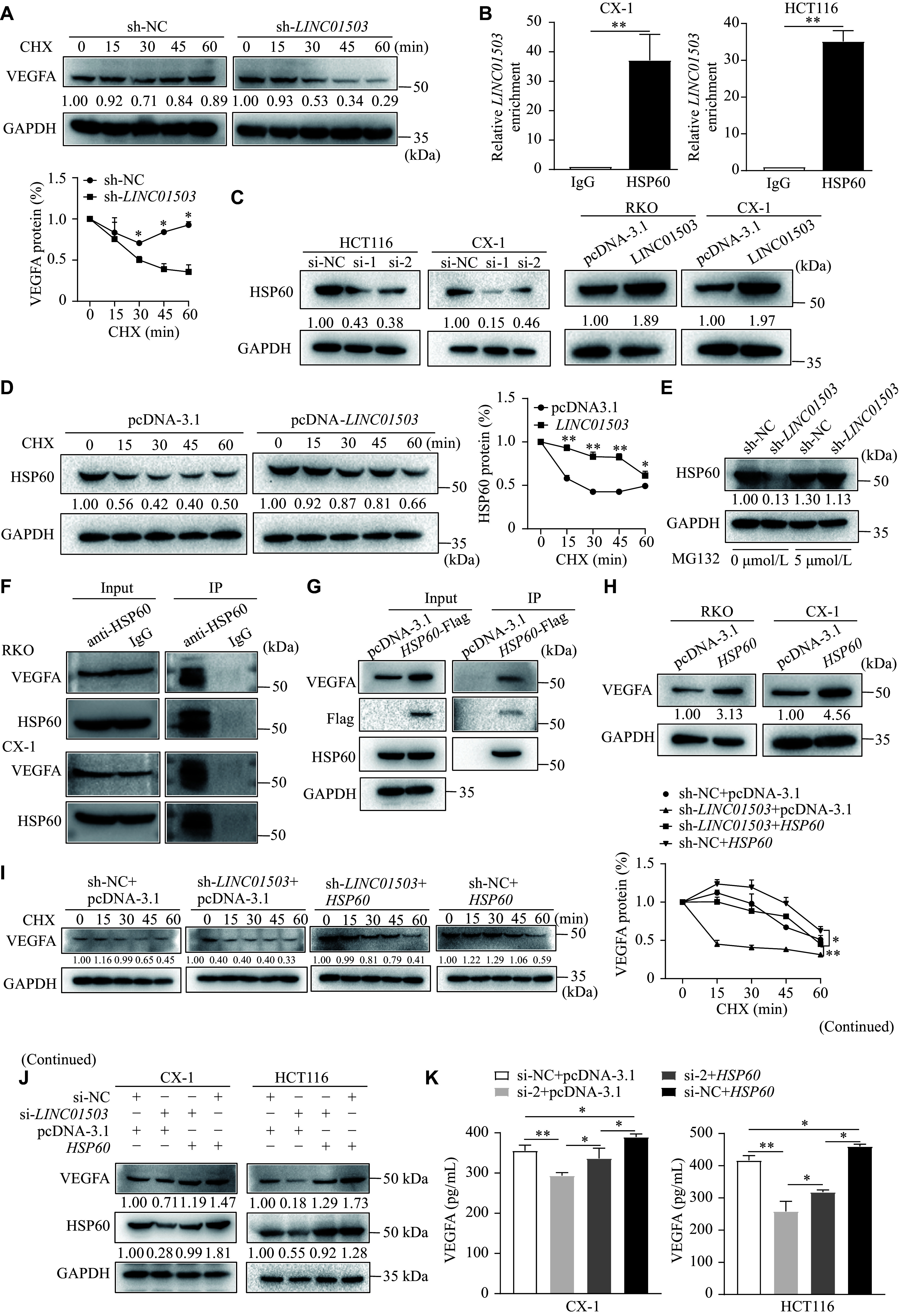
*LINC01503* enhanced the protein stability of VEGFA by binding to HSP60. A: Stable sh-*LINC01503* and negative control (sh-NC) HCT116 cells were treated with 100 μg/mL cycloheximide (CHX) and harvested at the indicated time points. VEGFA protein levels were analyzed by Western blotting (WB), quantified by densitometry, and plotted against time to assess VEGFA stability. B: The RNA immunoprecipitation (RIP) assay was performed using anti-HSP60 antibody in HCT116 cells, with IgG as a negative control. Enrichment of *LINC01503* was quantified by qRT-PCR. C: WB was performed to measure HSP60 levels in *LINC01503* knockdown HCT116 and CX-1 cells, as well as in *LINC01503*-overexpressing RKO and CX-1 cells. D: HCT116 cells transfected with *LINC01503*-overexpression plasmid or pcDNA-3.1 were treated with 100 μg/mL CHX, and proteins were harvested at the indicated time points. HSP60 protein levels were detected by WB and plotted against time to evaluate HSP60 stability. E: Stable sh-*LINC01503* and sh-NC HCT116 cells were treated with 5 μmol/L proteasome inhibitor (MG132) for 12 h, and HSP60 protein levels were measured by WB. F and G: Immunoprecipitation was performed using anti-HSP60 (F) and anti-Flag (G) antibodies to investigate the interaction between HSP60 and VEGFA in HCT116 cells. H: WB was used to quantify VEGFA levels in HSP60-overexpressing RKO and CX-1 cells. I: HSP60 overexpression plasmid (HSP60) or control plasmid (pcDNA3.1) was transfected into *LINC01503*-knockdown stable HCT116 cells, followed by treatment with 100 μg/mL CHX at various time points after 48 h of transfection; VEGFA protein levels were detected by WB and plotted against time to assess stability. J and K: CX-1 and RKO cells were co-transfected with si-*LINC01503* and *HSP60* overexpression plasmid, with VEGFA levels in cell lysates evaluated by WB (J) and secreted VEGFA levels assessed by ELISA (K). Data are presented as mean ± standard deviation (*n* = 3). Statistical significance was evaluated using one-way ANOVA, followed by Dunnett's test for multiple comparisons and unpaired Student's *t*-test. ^*^P < 0.05 and ^**^P < 0.01. Abbreviations: CRC, colorectal cancer; qRT-PCR, real-time reverse transcription-PCR; VEGFA, vascular endothelial growth factor A.

We next demonstrated that, under cycloheximide (CHX) treatment, knocking down *LINC01503* in CRC cells significantly enhanced VEGFA protein degradation, compared with the control group (***Fig. 7A***). Several studies have shown that lncRNAs may affect protein stability by binding to proteins^[26]^. Therefore, we hypothesized that *LINC01503* regulated the protein stability of VEGFA by binding to specific proteins.

To test this, we performed ChIRP assays using biotin-labeled probes specific for *LINC01503* (***Supplementary Fig. 4A***, available online), before subjecting these samples to mass spectrometry analysis. While *LINC01503* did not directly bind to VEGFA, we found that it interacted with the HSP60 chaperone, and we next selected this protein for further study (***Supplementary Fig. 4B***, available online). The RIP assays demonstrated that *LINC01503* directly bound to HSP60 (***Fig. 7B***). Moreover, *LINC01503* knockdown decreased HSP60 expression, while *LINC01503* overexpression increased HSP60 levels (***Fig. 7C***). However, *LINC01503* knockdown or overexpression did not significantly affect *HSP60* mRNA levels (***Supplementary Fig. 4C***, available online), indicating that *LINC01503* may instead affect HSP60 protein stability. To verify this, we examined the stability of HSP60 in CRC cells following CHX treatment, and found that overexpressing *LINC01503* in CHX-treated CRC cells reduced the degradation rate of HSP60 protein (***Fig. 7D***). Additionally, the results of the MG132 treatment experiments showed that *LINC01503* affected HSP60 stability through a proteasome-dependent degradation pathway (***Fig. 7E***). These results indicate that *LINC01503* may increase HSP60 levels by inhibiting its degradation *via* the proteasomal degradation pathway.

As a chaperone, HSP60 binds to proteins to increase their stability^[27]^. We therefore speculated that the stabilizing effect of *LINC01503* on the VEGFA protein might be mediated by HSP60. To validate this hypothesis, we first performed the IP and Co-IP experiments using the anti-HSP60 and anti-Flag antibodies, and found that HSP60 bound to VEGFA (***Fig. 7F*** and ***7G***). We then examined the effects of HSP60 on VEGFA protein levels, and found that high HSP60 expression significantly increased the protein levels of VEGFA (***Fig. 7H***). Meanwhile, HSP60 rescued VEGFA protein levels from *LINC01503*-knockdown-mediated degradation (***Fig. 7I***), indicating that HSP60 increased VEGFA protein stability. Rescue experiments also showed that high HSP60 expression restored the expression and secretion of VEGFA in CRC cells following *LINC01503* silencing (***Fig. 7J*** and ***7K***). Finally, HSP60 reversed the inhibitory effect of *LINC01503* downregulation in CRC cells on the tube formation and migration ability of HUVECs (***Supplementary Fig. 4C***–***4E***, available online).

Taken together, our results suggest that *LINC01503* may increase the stability of VEGFA by binding to HSP60, thereby promoting the growth and metastasis of vascular endothelial cells.

### *LINC01503* was transcriptionally activated by H3K27ac

**Figure 8:**
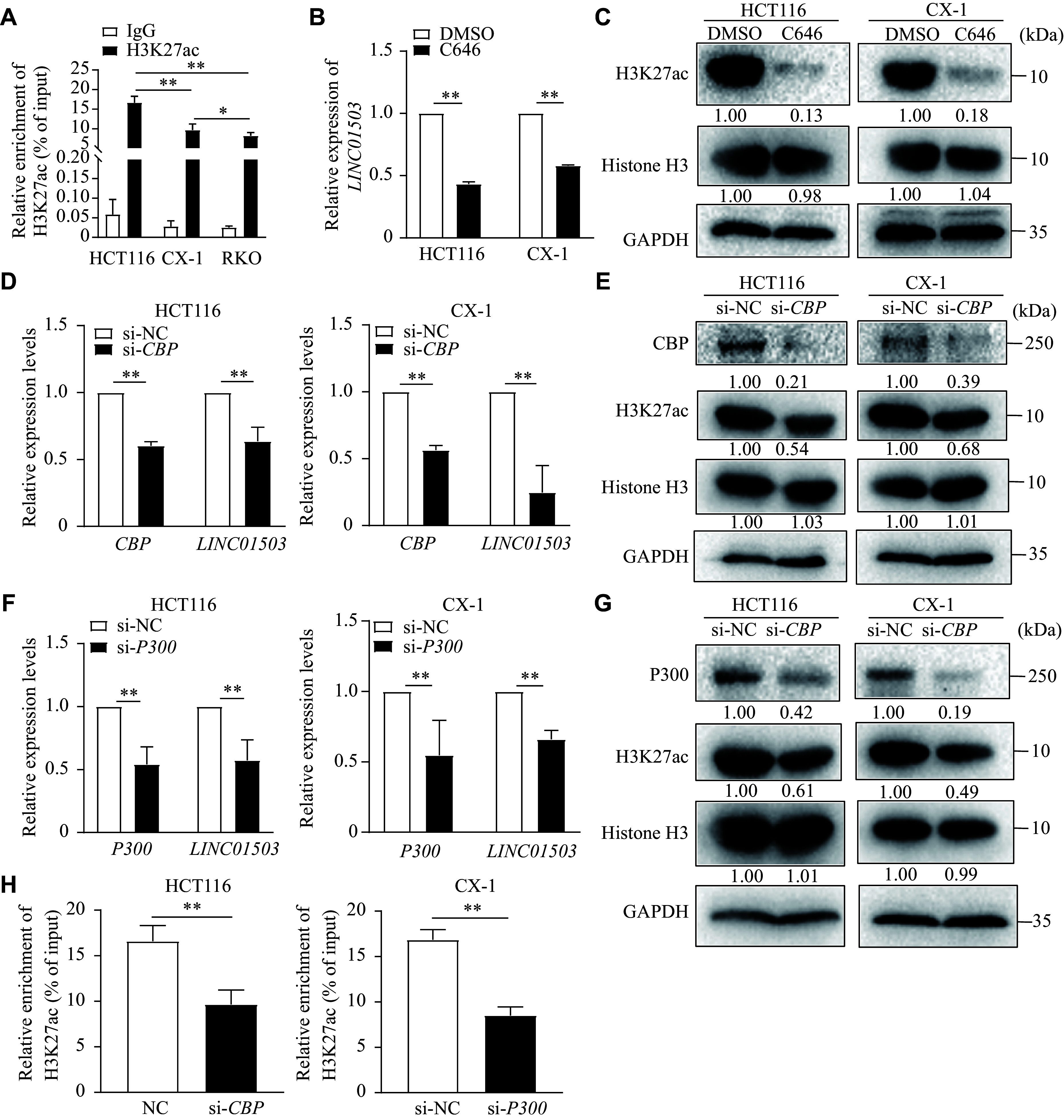
*LINC01503* was transcriptionally activated by histone H3 lysine 27 (H3K27) acetylation. A: Chromatin immunoprecipitation (ChIP) followed by qRT-PCR was performed to assess H3K27ac enrichment in various CRC cells with differing *LINC01503* levels. B and C: HCT116 and CX-1 cells were treated with C646 (20 μmol/L) or DMSO for 48 h. *LINC01503* expression levels were detected by qRT-PCR (B). Protein levels of H3K27ac and total H3 were analyzed by Western blotting (WB; C). D–G: HCT116 and CX-1 cells were transfected with specific siRNAs targeting *CBP* and *P300*. After 24 h, the mRNA levels of *CBP*, *P300*, and *LINC01503* were quantified by qRT-PCR (D and F); and protein levels of H3K27ac and total H3 were measured by WB (E and G). H: Following *CBP* or *P300* siRNA transfection in HCT116 cells, the ChIP assay combined with qRT-PCR was used to detect H3K27ac enrichment within the *LINC01503* promoter region. Data are presented as mean ± standard deviation (*n* = 3). ^*^P < 0.05 and ^**^P < 0.01 by one-way ANOVA, followed by Dunnett's tests for multiple comparisons and unpaired Student's *t*-test. Abbreviations: CRC, colorectal cancer; qRT-PCR, real-time reverse transcription-PCR.

Increased levels of H3K27ac are commonly found in CRC versus normal tissues^[28]^. To investigate whether the increased expression of *LINC01503* in CRC cell lines was related to abnormal histone modifications, we conducted a genomic bioinformatics analysis using the UCSC Genome Browser (http://genome.ucsc.edu/), and found a significant enrichment of the H3K27ac peak in the promoter region of *LINC01503* (***Supplementary Fig. 5A***, available online), indicating that *LINC01503* transcription might be regulated by H3K27ac modification. We further assessed the enrichment of H3K27ac in the *LINC01503* promoter across various CRC cell lines with differing *LINC01503* expression levels. The results showed that CRC cells with higher H3K27ac enrichment exhibited correspondingly higher *LINC01503* expression levels (***Fig. 8A***, ***Supplementary Fig. 5B*** and ***5C*** [available online]). The H3K27ac modification is mediated by the CREB-binding protein (CBP)/p300 complex^[29]^. C646, a histone acetyltransferase inhibitor targeting p300, significantly reduced H3K27ac and *LINC01503* levels in CRC cells (***Fig. 8B*** and ***8C***). Moreover, transfecting CRC cell lines with siRNAs targeting CBP or p300 led to a reduction in H3K27ac and *LINC01503* levels (***Fig. 8D***–***8G***). Furthermore, knocking down CBP or p300 significantly reduced the enrichment of H3K27ac in the promoter region of *LINC01503* (***Fig. 8H***). These results indicate that the expression of *LINC01503* may be regulated by the H3K27ac modification.

**Figure 9:**
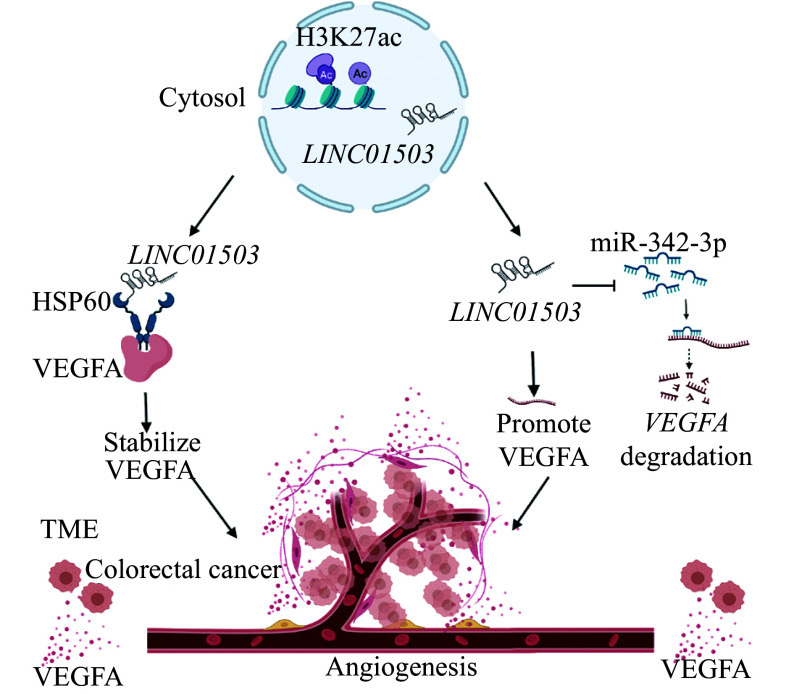
The proposed mechanism by which *LINC01503* promotes angiogenesis in colorectal cancer. *LINC01503*, which is transcriptionally regulated by histone acetylation, promotes angiogenesis in colorectal cancer by inducing the expression of VEGFA through both the miR-342-3p/VEGFA and HSP60/VEGFA signaling pathways. Abbreviations: VEGFA, vascular endothelial growth factor A; H3K27ac, histone H3 acetylated lysine 27; HSP60, heat shock protein family 60; TME, tumor microenvironment.

Collectively, these findings suggest that the H3K27ac-mediated transcriptional regulation of *LINC01503* promotes angiogenesis in CRC by inducing VEGFA expression in a miR-342-3p and HSP60-dependent manner (***Fig. 9***).

## Discussion

In the current study, we demonstrated that elevated *LINC01503* expression in CRC tumor tissues was relevant to tumor progression, vascular invasion, clinical stage, and poor prognosis. These findings implied that *LINC01503* might be an independent prognostic marker for CRC. Additionally, *LINC01503* expression was positively correlated with CD31 and VEGFA levels. Functional assays, both *in vitro* and *in vivo*, demonstrated that *LINC01503* enhanced the proliferation, migration, and invasion of CRC cells. Furthermore, *LINC01503* was found to promote angiogenesis by upregulating VEGFA expression. Collectively, these results indicate that *LINC01503* may contribute to CRC development through the promotion of angiogenesis.

Under physiological conditions, angiogenesis is delicately regulated by pro-angiogenic and anti-angiogenic regulatory factors. In tumor tissues, however, this balance is disrupted, resulting in increased angiogenesis^[30]^. Vascular endothelial cell function within the tumor microenvironment is controlled by VEGF, a cytokine secreted by tumor cells. In addition, other angiogenic signaling pathways supply tumors with oxygen and nutrients to maintain tumor cell proliferation; this environment also creates some favorable conditions for tumor metastasis^[8]^. Many studies have shown that lncRNAs affect tumor progression by regulating angiogenesis in CRC. For instance, lncRNA *SNHG17* accelerated CRC cell proliferation and migration by sponging miR-23a-3p to regulate CXCL12-mediated angiogenesis^[31]^. The levels of angiogenesis regulators HIF-1α and VEGF were increased in CRC cells with high lncRNA *SH3pXD2A-AS1* expression^[32]^. LncRNA *ZFAS1* upregulated the expression of VEGFA through ceRNA^[33]^, while lncRNA *TPT1-AS1* promoted the secretion of VEGFA, which played a key role in CRC angiogenesis^[9]^. These studies provide a theoretical basis for lncRNA as targets in anti-angiogenic cancer therapy.

In the current study, we found that *LINC01503* promoted VEGFA expression by binding to miR-342-3p, thereby increasing the tube formation and migration ability of HUVECs. In addition to the regulation of VEGFA at the mRNA levels, we found that *LINC01503* enhanced VEGFA protein stability. Through ChIRP and mass spectrometry analyses, we identified HSP60 as a binding partner of *LINC01503*, and RIP assays demonstrated the direct interaction between HSP60 and *LINC01503*. Further experiments demonstrated that *LINC01503* promoted HSP60 protein expression by inhibiting its ubiquitin-dependent degradation. Moreover, HSP60 was shown to bind and stabilize VEGFA protein. Rescue experiments further showed that *LINC01503* stabilized VEGFA protein by binding to HSP60, thereby promoting the growth and metastasis of vascular endothelial cells.

Heat shock proteins (HSPs) are molecular chaperones, which exert their biological functions by affecting protein stability^[34]^. HSPs such as HSP60, HSP70, HSP90, and HSP110 are widely and highly expressed in CRC, liver cancer, and other cancers^[34–35]^. High HSP expression may contribute to the establishment of an immunosuppressive tumor microenvironment, and it may promote tumorigenesis, poor prognosis, and treatment resistance^[34,36]^. HSPs also exert pro-cancer effects by maintaining the stability and functional integrity of angiogenic and oncogenic proteins^[36–37]^. For instance, HSP90 promoted tumor angiogenesis and growth by binding to and stabilizing protein kinase D2^[38]^ and macrophage migration inhibitory factor^[39]^. Therefore, the regulation of HSP60 by *LINC01503* may be a critical step in preventing the aberrant stabilization of major pro-angiogenic oncogenic proteins.

CRC tumors induce neovascularization, which provides a strong rationale for antiangiogenic therapies. Indeed, anti-VEGF therapy has already been approved by the FDA for the treatment of metastatic CRC^[40]^. However, its clinical application is limited because of fatal bleeding, intestinal perforation, and arterial embolism. Additionally, the mechanisms of adaptive anti-angiogenic therapy, particularly evasion of VEGF inhibition, remain poorly understood^[41–42]^. LncRNAs have been recently reported to be involved in tumor progression by regulating the expression of VEGF in metastatic CRC^[17–18]^. Therefore, the search for upstream regulatory molecules of VEGF, such as LINC01503, may provide novel targets for the development of more effective and safer anti-VEGF therapeutic strategies. In the current study, we found that *LINC01503* promoted the expression of VEGFA by simultaneously regulating both mRNA and protein stability of VEGFA. Targeted silencing of *LINC01503* not only reduced the expression of VEGFA in CRC cells, but it also significantly inhibited tumor growth and metastasis of transplanted tumors in nude mice. Moreover, histone acetylation in tumors has previously been shown to promote VEGFA transcription^[43]^. Similarly, our results demonstrated that histone acetylation also promoted *LINC01503* transcription. These results suggest that histone acetylation may, on the one hand, upregulate VEGFA by promoting its RNA level, and on the other hand, may also increase the expression levels of *LINC01503* epigenetically, thereby upregulating VEGFA expression through *LINC01503*. These findings imply that *LINC01503* may potentially be targeted in the anti-angiogenic therapy and serve as a VEGFA regulatory molecule to evaluate the effect of an anti-VEGF therapy.

Although there are some important findings from our current study, there are also some limitations. We lack enough evidence to confirm whether the oncogenic role of *LINC01503* in CRC mainly depends on VEGFA protein. Besides the epigenetic regulation of *LINC01503*, we also need to investigate whether some small molecules, such as vitamin D, can be used to regulate its expression^[44–45]^, which will also be the focus of our future research.

In summary, the current study demonstrates that *LINC01503* may regulate VEGFA expression through the miR-342-3p/VEGFA and HSP60/VEGFA axes, activate the AKT signaling pathway downstream of VEGFA in vascular endothelial cells in a paracrine manner, and promote angiogenesis, which in turn drives CRC progression.

## Fundings

This work was supported by the National Natural Science Foundation of China (Grant No. 81972288) and the Scientific and Technologic Development Programme of Suzhou (Livelihood Science and Technology-Applied Basic Research in Healthcare, SYS2020057).

## SUPPLEMENTARY DATA

Supplementary data to this article can be found online.
