# Supplementary material for: LncRNA LINC01503 promotes angiogenesis in colorectal cancer by regulating VEGFA expression via miR-342-3p and HSP60 binding
Source: J Biomed Res. 2024 Oct 22;39(3):286–304. doi: 10.7555/JBR.38.20240190 (PMC12239982; doi:10.7555/JBR.38.20240190)
Supplement: Supplementary file 1 — Supplementary data to this article can be found online. [file jbr-39-3-286-Supplementary.pdf]

Available online at www.jbr-pub.org.cn

Open Access at PubMed Central

Journal of Biomedical Research, 2025 39(3): 1–6

Supplementary Data

JBR

LncRNA *LINC01503* promotes angiogenesis in colorectal cancer by regulating VEGFA expression *via* miR-342-3p and HSP60 binding

Dandan Zheng[1,△], Xiya Zhang[2,△], Jia Xu[1], Shuwen Chen[3], Bin Wang[4], Xiaoqin Yuan[1,✉]

[1]*Department of Anatomy, Histology and Embryology, Nanjing Medical University, Nanjing, Jiangsu 211166, China;*
[2]*Jiangsu Key Laboratory of Oral Diseases, Nanjing Medical University, Nanjing, Jiangsu 210029, China;*
[3]*Department of Clinical Medicine, First Clinical Medicine College, Nanjing Medical University, Nanjing, Jiangsu 210029, China;*
[4]*Department of Gastroenterology, the Affiliated Changshu Hospital of Nantong University, Changshu No.2 People's Hospital, Suzhou, Jiangsu 215500, China.*

Supplementary Table 1 **Sequence of siRNAs**

siRNAs	Sequences (5′-3′)
si-*LINC01503*-1	GGAGACAAATGACGGCCTT
si-*LINC01503*-2	CCACCTTTCTGGTAATGCA
si-*LINC01503*-3	CCAAAGCTCTGTTATTAAA
si-*CBP*	GGAGCCAUCUAGUGCAUAATT
si-*P300*	AACCCCUCCUCUUCAGCACCATT

Supplementary Table 2 **Primers used in qRT-PCR**

Genes	Sequences (5′-3′)
GAPDH-forward	GGAGCGAGATCCCTCCAAAAT
GAPDH-reverse	GGCTGTTGTCATACTTCTCATGG
U6-forward	CTCGCTTCGGCAGCACA
U6-reverse	AACGCTTCACGAATTTGCGT
LINC01503-forward	CCAGCCGAATACTCTTGCAC
LINC01503-reverse	ATGCCTTAAACACCCATCCG
VEGFA-forward	AGGGCAGAATCATCACGAAGT
VEGFA-reverse	AGGGTCTCGATTGGATGGCA
VEGFB-forward	GAGATGTCCCTGGAAGAACACA
VEGFB-reverse	GAGTGGGATGGGTGATGTCAG
EPO-forward	GGAGGCCGAGAATATCACGAC
EPO-reverse	CCCTGCCAGACTTCTACGG
PDGF-forward	TTGTACCGAAGAGATGAGACCA
PDGF-reverse	GCTGTATCCGTGTATTCTCCTGA

Supplementary Table 3 **Primers used in CHIP assay**

Genes	Sequences (5′-3′)
LINC01503-1-forward	GGACTTAGAGGAACTCCGGC
LINC01503-1-reverse	CAGAACCTCGGTACCCACAG
LINC01503-2-forward	ATGATCTCTCAGCCCCTCCC
LINC01503-2-reverse	GGTTCTTCCTCCTGGGTTGG
LINC01503-3-forward	CTGCACGTCTTGGTTGCTCT
LINC01503-3-reverse	TGGCAGCAGGCATGAAAATC
LINC01503-4-forward	GCACGTCTTGGTTGCTCTCA
LINC01503-4-reverse	CATGACTCACCCAGCAGACA
LINC01503-5-forward	TTCCTGTAACTCGGACCCCT
LINC01503-5-reverse	CTATCTGGGCCTCTGTCCCT
LINC01503-6-forward	CACAGCAGGGAGGTCACATT
LINC01503-6-reverse	TGGGAAGTGGTGGGGAGTTA
LINC01503-7-forward	AATGAATGAAGGCCAAACTTGAG
LINC01503-7-reverse	TTGAAAAGACCCAGAGGAAGAGG

△These authors contributed equally to this work.

✉Corresponding author: Xiaoqin Yuan, Department of Anatomy, Histology and Embryology, Nanjing Medical University, 101 Longmian Avenue, Jiangning District, Nanjing, Jiangsu 211166, China. E-mail: yuanxq@njmu.edu.cn.

Received: 05 July 2024; Revised: 11 October 2024; Accepted: 12 October 2024; Published online: 22 October 2024

CLC number: R735.3, Document code: A

The authors reported no conflict of interests.

This is an open access article under the Creative Commons Attribution (CC BY 4.0) license, which permits others to distribute, remix, adapt and build upon this work, for commercial use, provided the original work is properly cited.

© 2025 by Journal of Biomedical Research.

https://doi.org/10.7555/JBR.38.20240190

Supplementary Table 4 **Primers and probes used in ChIRP assay**

Genes	Sequences (5′-3′)
LINC01503-1-forward	CCAGCCGAATACTCTTGCAC
LINC01503-1-reverse	ATGCCTTAAACACCCATCCG
LINC01503-2-forward	GCCTGACACGTAGGTACACA
LINC01503-2-reverse	GGGCCGCTCTTGGATTTTCA
LINC01503-3-forward	GGAAGGATGGGCCTGACAC
LINC01503-3-reverse	CGCTCTTGGATTTTCATGCCT
GAPDH-F-forward	GGAGCGAGATCCCTCCAAAAT
GAPDH-F-reverse	GGCTGTTGTCATACTTCTCATGG
p*LINC01503*-1	AGAAAGGTGGGTATTCCGAG
p*LINC01503*-2	GTAGGTACACACTTGTCAGA
p*LINC01503*-3	ACACATCAGCATCACTGTTG
p*LINC01503*-4	GGGAGGGTGTATTCAGAGAG
p*LINC01503*-5	CAAGAGTATTCGGCTGGTAA
p*LINC01503*-6	TGCTGAAAGAAACTCATTGC
pCTL	TTCTCCGAACGTGTCACGT

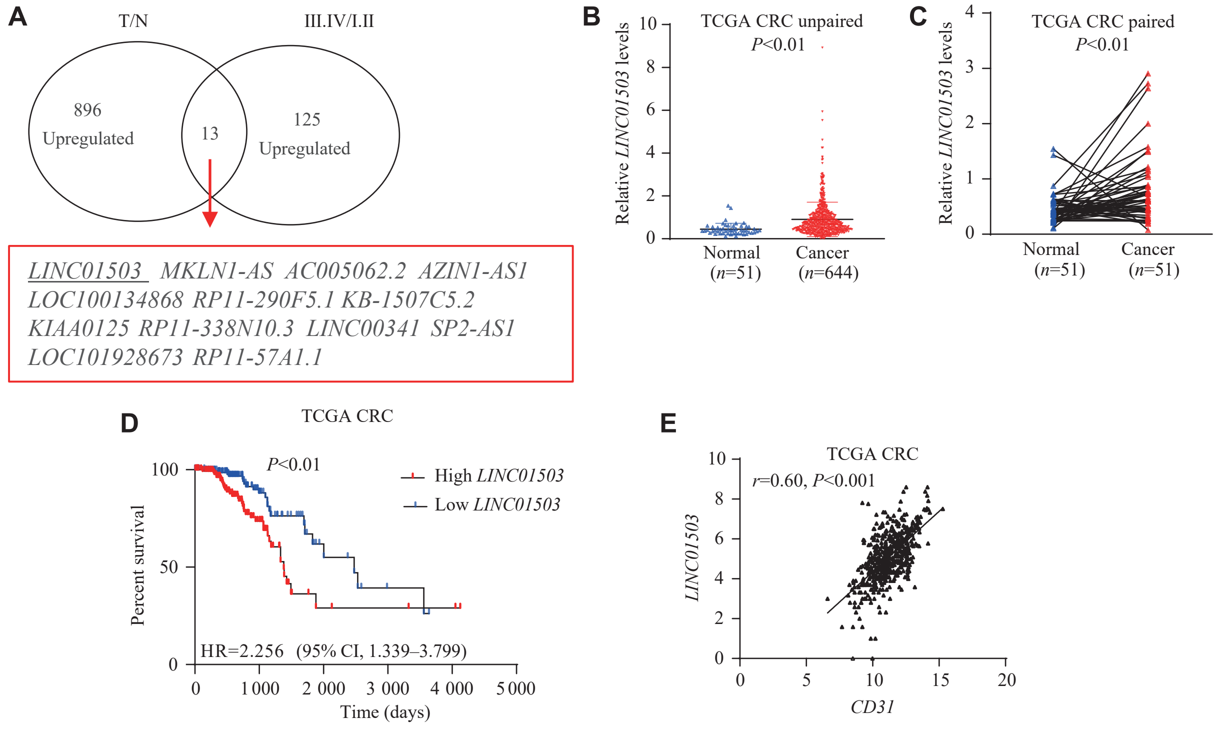

Supplementary Fig. 1 ***LINC01503*** **was overexpressed in colorectal cancer (CRC) tissues and was associated with poor prognosis in TCGA.** A: Based on previous microarray results, genes upregulated in tumor (T) versus normal (N) tissues were intersected with those upregulated in stage Ⅲ/Ⅳ versus stage Ⅰ/Ⅱ tumors, resulting in the identification of 13 candidate genes. B: The relative expression levels of *LINC01503* in colorectal cancer (CRC) tissues were analyzed using data from The Cancer Genome Atlas (TCGA), by comparing CRC tissues with normal tissues. Statistical significance was assessed using an unpaired Student's *t*-test ($P < 0.01$). C: The expression levels of *LINC01503* in paired CRC samples and their corresponding normal tissues from the TCGA database were analyzed, with significance determined by a paired Student's *t*-test ($P < 0.01$). D: Kaplan-Meier survival curve analysis for CRC patients stratified by low versus high *LINC01503* expression levels using TCGA data, with the *P*-value calculated using the log-rank test. E: Correlation analysis between *LINC01503* and *CD31* mRNA expression levels in the TCGA database.

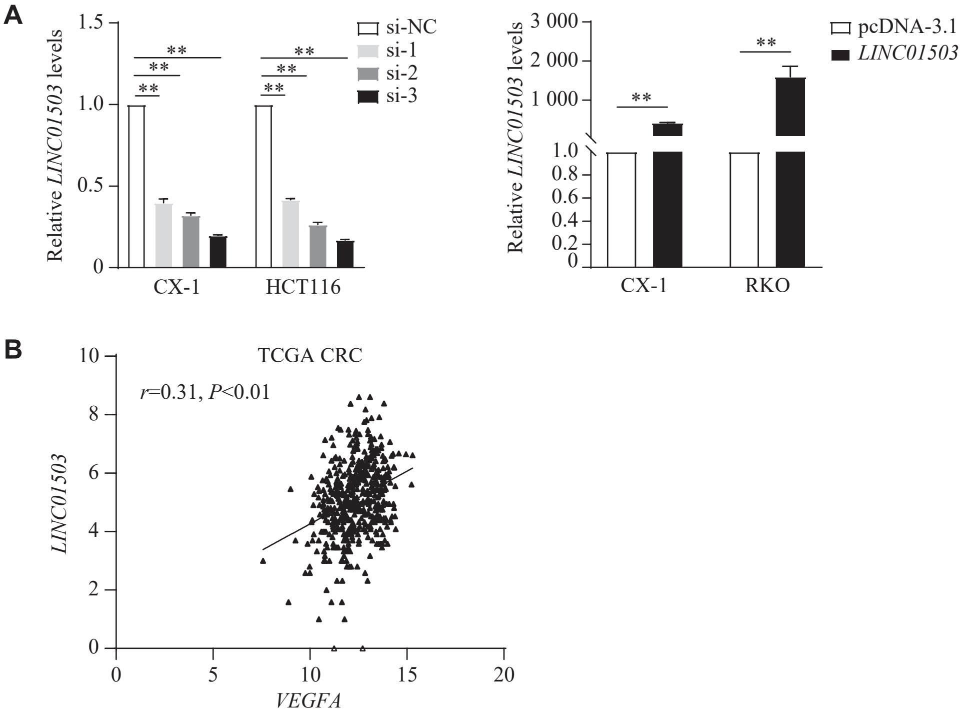

***Supplementary Fig. 2 LINC01503* expression was associated with VEGFA levels.** A: The transfection efficiency of *LINC01503* siRNAs in HCT116 and CX-1 cells (left) and that of overexpression plasmid in CX-1 and RKO cells (right) was assessed by real-time reverse transcription-PCR, with *ACTB* serving as an internal reference gene. B: Correlation analysis of *LINC01503* and *VEGFA* mRNA expression levels in the TCGA database. Data are presented as mean ± standard deviation from three independent experiments. $^{**}P < 0.01$, analyzed by one-way ANOVA followed by Dunnett's tests for multiple comparisons and unpaired Student's *t*-test.

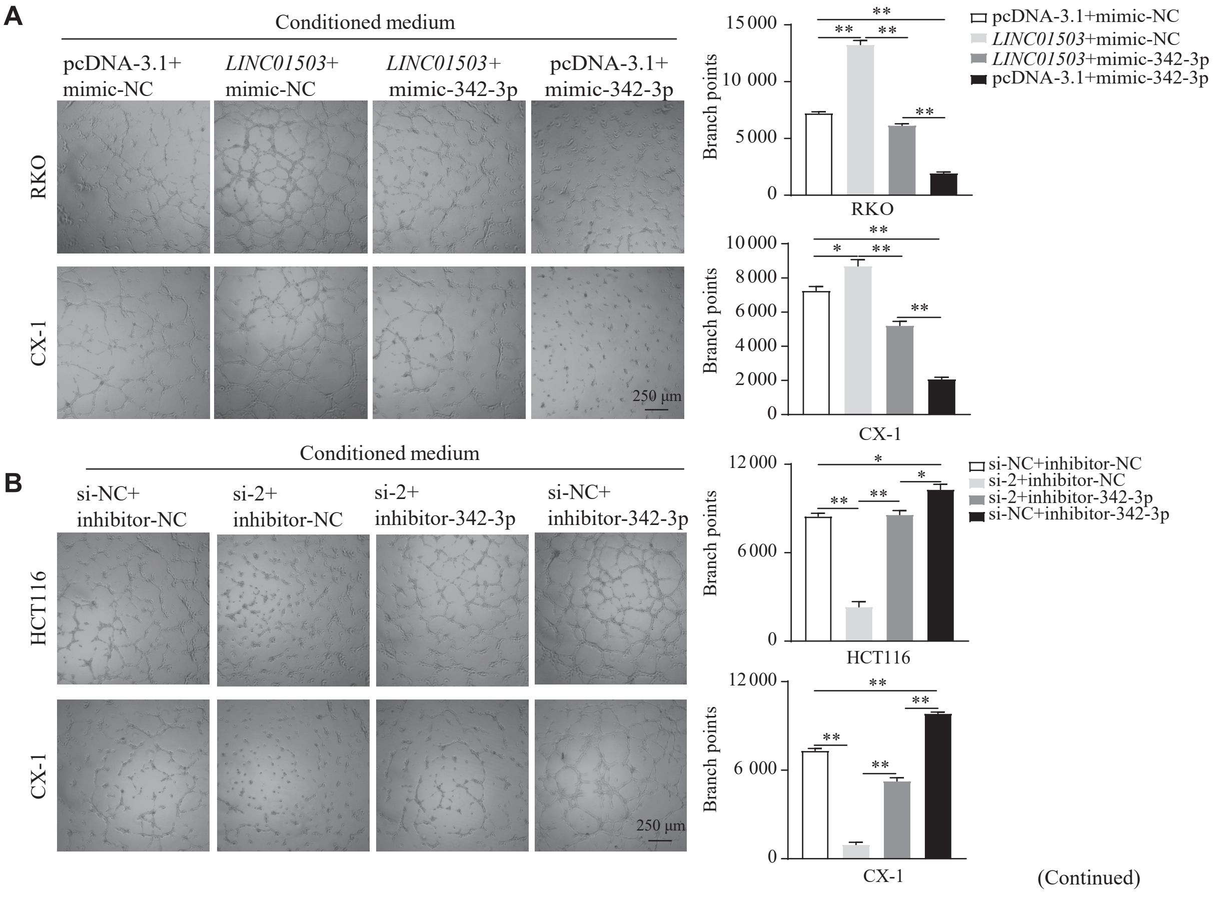

(Continued)

(Continued)

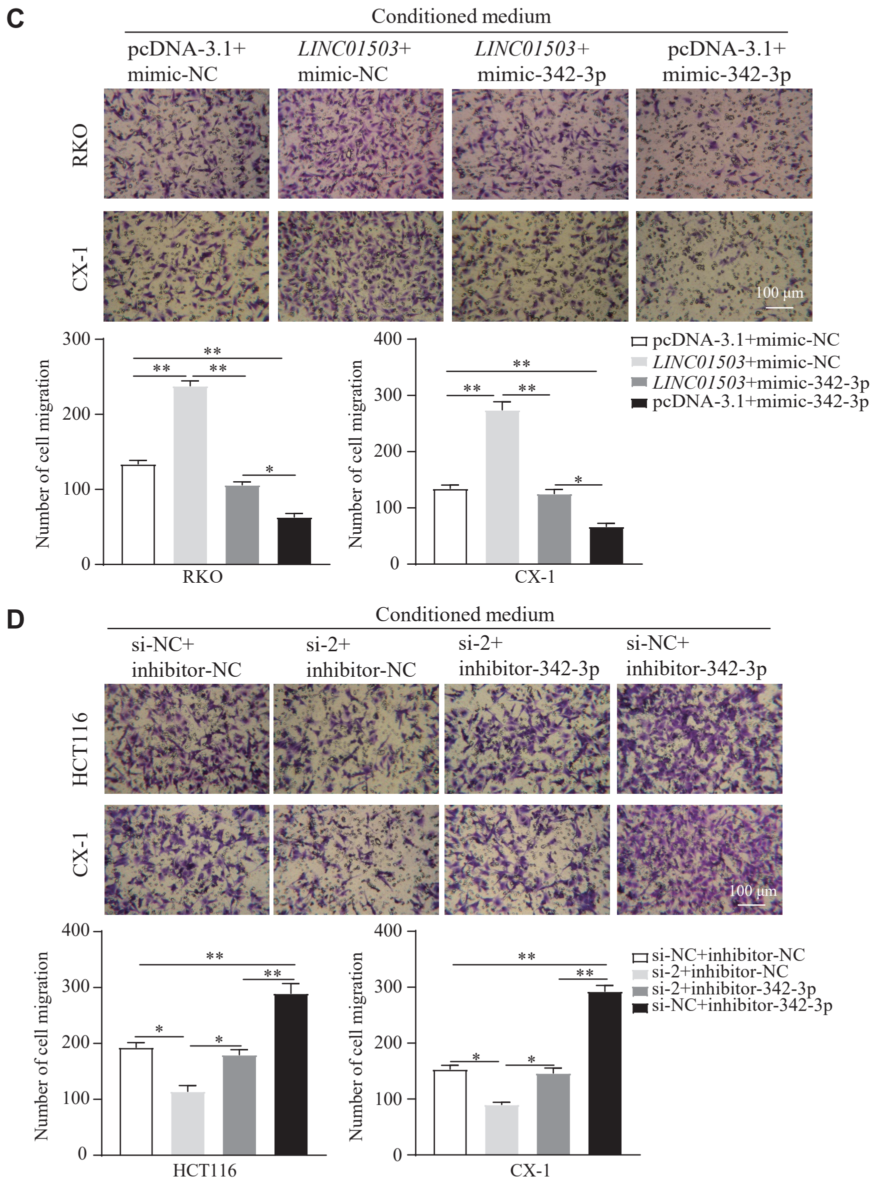

Supplementary Fig. 3 ***LINC01503*** **promoted VEGFA expression by binding to miR-342-3p.** A and B: *LINC01503* overexpression plasmid and miR-342-3p mimics were co-transfected into RKO and CX-1 cells (A), and *LINC01503* siRNA and miR-342-3p inhibitor were co-transfected into HCT116 and CX-1 cells (B). After 48 h, the serum-free medium was replaced, and the conditioned medium was harvested after an additional 48-h culture period. The conditioned medium was used to treat human umbilical vein endothelial cells for 6 h to examine tube formation, with the total tube length quantified. C and D: After co-transfection of *LINC01503* overexpression plasmid and miR-342-3p mimics in RKO and CX-1 cells (C) or co-transfection of *LINC01503* siRNA and miR-342-3p inhibitor in HCT116 and CX-1 cells (D) for 48 h, conditioned medium was collected as mentioned above, and was used to treat HUVECs for 24 h, with migration capacity examined using a transwell assay. Data are presented as mean ± standard deviation from three independent experiments. $^{*}P < 0.05$ and $^{**}P < 0.01$ by one-way ANOVA, followed by Dunnett's tests for multiple comparisons and unpaired Student's t-test.

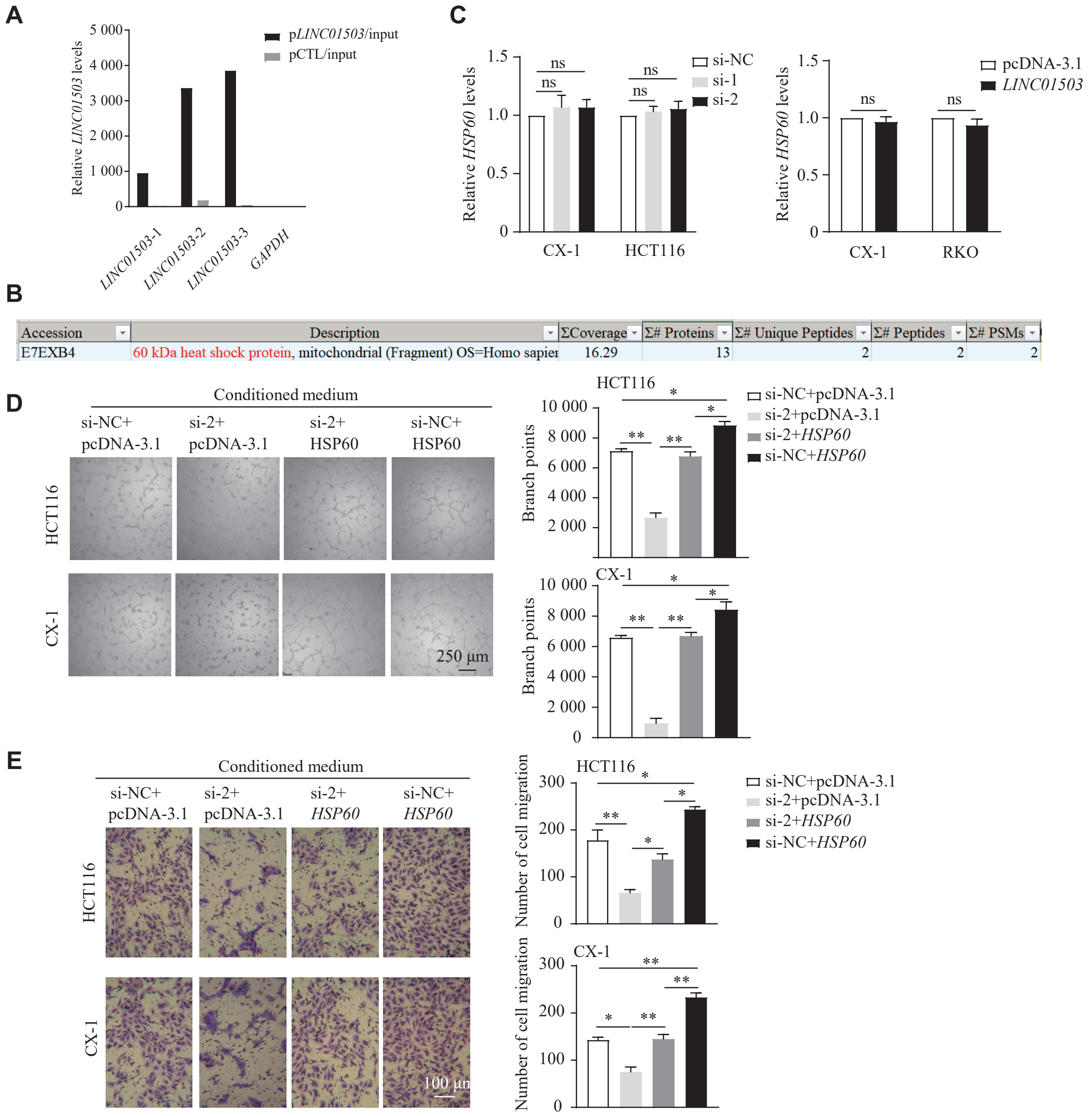

Supplementary Fig. 4 ***LINC01503*** **promoted VEGFA expression by binding to HSP60.** A: *LINC01503* binding proteins were precipitated using chromatin isolation by RNA purification assay in HCT116 cells. Relative *LINC01503* levels were examined using targeted and control probes. B: HSP60 was subsequently identified by mass spectrometry. C: *HSP60* mRNA expression levels in *LINC01503* knockdown HCT116 and CX-1 cells, as well as in *LINC01503* overexpressing RKO and CX-1 cells, were examined using real-time reverse transcription-PCR. D and E: After 48 h of transfection with *LINC01503* siRNA and *HSP60* overexpression plasmid in CX-1 and HCT116 cells, the serum-free medium was replaced. Conditioned medium was collected after 24 h and used to treat human umbilical vein endothelial cells (HUVECs) to measure tube formation (D) and migration (E). Data are presented as mean ± standard deviation from three independent experiments. $^{*}P < 0.05$ and $^{**}P < 0.01$, analyzed by one-way ANOVA followed by Dunnett's tests for multiple comparisons and unpaired Student's *t*-test.

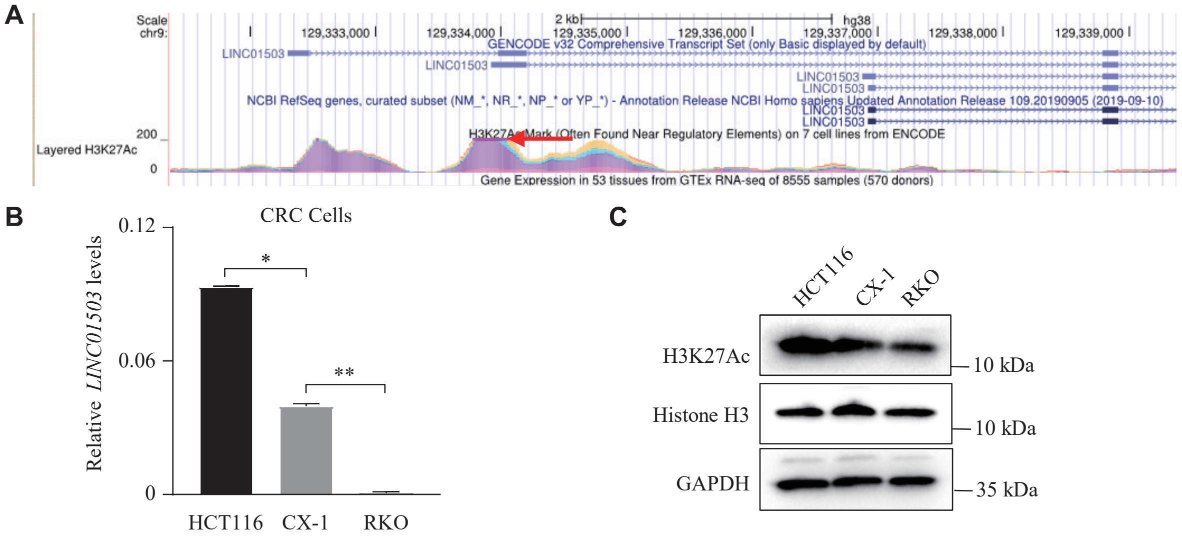

Supplementary Fig. 5 **H3K27ac in *LINC01503* promoter were varied in different colorectal cancer cells.** A: Genomic bioinformatics analysis showed the enrichment of H3K27ac in *LINC01503* promoter (indicated by the red arrow). The data were sourced from http://genome.ucsc.edu/. B: *LINC01503* expression levels in HCT116, CX-1, and RKO cells were examined by real-time reverse transcription-PCR. C: Protein levels of H3K27ac and total histone-3 were assessed by Western blotting in various colorectal cancer cell lines. Data are presented as mean ± standard deviation from three independent experiments. $^{*}P < 0.05$ and $^{**}P < 0.01$ by one-way ANOVA, followed by Dunnett's tests for multiple comparisons and unpaired Student's *t*-test.
